# *KLF4*, a Key Regulator of a Transitive Triplet, Acts on the TGF-β Signaling Pathway and Contributes to High-Altitude Adaptation of Tibetan Pigs

**DOI:** 10.3389/fgene.2021.628192

**Published:** 2021-04-15

**Authors:** Tao Wang, Yuanyuan Guo, Shengwei Liu, Chaoxin Zhang, Tongyan Cui, Kun Ding, Peng Wang, Xibiao Wang, Zhipeng Wang

**Affiliations:** ^1^College of Animal Science and Technology, Northeast Agricultural University, Harbin, China; ^2^Bioinformatics Center, Northeast Agricultural University, Harbin, China; ^3^College of Computer Science and Technology, Inner Mongolia Normal University, Hohhot, China; ^4^HeiLongJiang Provincial Husbandry Department, Harbin, China

**Keywords:** Tibetan pig, multitissue, transcriptome, hypoxia adaptation, gene network

## Abstract

Tibetan pigs are native mammalian species on the Tibetan Plateau that have evolved distinct physiological traits that allow them to tolerate high-altitude hypoxic environments. However, the genetic mechanism underlying this adaptation remains elusive. Here, based on multitissue transcriptional data from high-altitude Tibetan pigs and low-altitude Rongchang pigs, we performed a weighted correlation network analysis (WGCNA) and identified key modules related to these tissues. Complex network analysis and bioinformatics analysis were integrated to identify key genes and three-node network motifs. We found that among the six tissues (muscle, liver, heart, spleen, kidneys, and lungs), lung tissue may be the key organs for Tibetan pigs to adapt to hypoxic environment. In the lung tissue of Tibetan pigs, we identified *KLF4*, *BCL6B*, *EGR1*, *EPAS1*, *SMAD6*, *SMAD7*, *KDR*, *ATOH8*, and *CCN1* genes as potential regulators of hypoxia adaption. We found that *KLF4* and *EGR1* genes might simultaneously regulate the *BCL6B* gene, forming a *KLF4–EGR1–BCL6B* complex. This complex, dominated by *KLF4*, may enhance the hypoxia tolerance of Tibetan pigs by mediating the TGF-β signaling pathway. The complex may also affect the PI3K-Akt signaling pathway, which plays an important role in angiogenesis caused by hypoxia. Therefore, we postulate that the *KLF4–EGR1–BCL6B* complex may be beneficial for Tibetan pigs to survive better in the hypoxia environments. Although further molecular experiments and independent large-scale studies are needed to verify our findings, these findings may provide new details of the regulatory architecture of hypoxia-adaptive genes and are valuable for understanding the genetic mechanism of hypoxic adaptation in mammals.

## Introduction

Hypoxia is a significant environmental characteristic of high altitude, which exerts a marked impact on biological organisms and imposes extreme physiological challenges in mammals. The Tibetan pig was originally distributed at altitudes of 2,900–4,300 m in the Tibetan Plateau (Ai et al., 2014). Physiological studies showed that Tibetan pigs have evolved physiological adaptations to high-altitude hypoxia, such as a thicker alveolar septum with more highly developed capillaries (Ma et al., 2019) and a larger and strong heart (Li et al., 2013). Therefore, they represent a suitable animal model for exploring the molecular mechanism of hypoxia adaptation in high-altitude organisms.

With the development of sequencing technology, the majority of studies have explored the genetic basis of hypoxic adaptation in Tibetan pigs from the perspective of selection signals (Li et al., 2013, 2016; Ai et al., 2014; Huang et al., 2019; Ma et al., 2019; Shang et al., 2020) or by using differential expression analysis between differential conditional gene expression in one tissue based on the transcriptome (Jia et al., 2016; Zhang B. et al., 2017) up to the present. Although previous studies have identified the *EPAS1*, *HIF1A*, *EGLN1*, *RGCC KLF6*, *TGFB2*, *EGLN3*, and *ACE* genes related to hypoxia, these genes may only explain a minority of genetic variance due to the case of the missing heritability. Therefore, the most detailed solution to the missing heritability problem would involve identifying all causal genetic variants (Young, 2019) and exploring related gene networks that have facilitated high-altitude adaptation of Tibetan pigs.

The adaptation of Tibetan pigs to hypoxia is a very complex biological process that may involve multiple genes and transcriptional regulation among genes. The gene network provides a systemic view of gene regulation by the coordinated activity of multiple genes and regulatory factors and serves as a medium for understanding the mechanism of gene regulation (Narang et al., 2015). Based on the gene expression profile, a gene network was constructed by quantitative modeling, which can be used for rational design of molecular approaches to target specific biological processes (Nishio et al., 2008) and infer new biological functions (Cheng and Gerstein, 2012; McLeay et al., 2012). Although gene expression status cannot completely determine gene function, constructing gene network based on gene expression profile may be a feasible method to explore the mechanism of hypoxia adaptation. Moreover, the gene network cannot only intuitively elucidate the regulatory relationship between genes but also identify important hub genes. These hub genes represent candidates for further experimental investigation and potential biomarkers for complex traits (Döhr et al., 2005; Buckingham and Rigby, 2014; Chen et al., 2016).

Transcription factors (TFs) and microRNAs (miRNAs) regulate gene expression at the transcriptional and posttranscriptional levels, respectively. They coordinately control the dynamics and output of gene transcription and tightly control spatial and temporal patterns of gene expression. Therefore, constructing a gene regulatory network involving TFs and miRNAs is helpful in understanding the regulatory mechanism of genes in adaptation to hypoxia.

**FIGURE 1:**
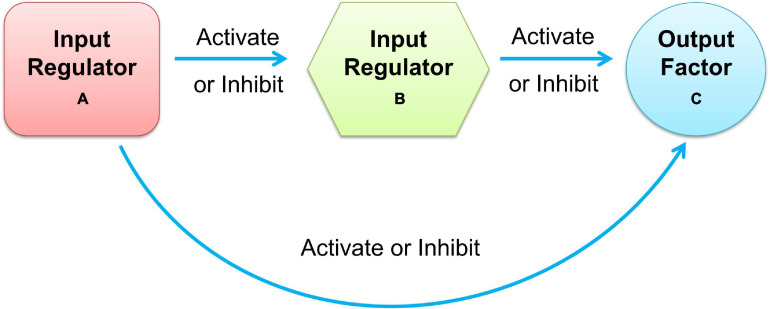
Schematic diagram of feedforward loop.

Moreover, most cellular tasks are not performed by individual genes but by groups of functionally associated genes, generally referred to as modules. In a gene regulatory network, modules appear as groups of densely interconnected nodes, also called communities or clusters (Adamcsek et al., 2006). Among these clusters of gene regulatory networks, size-3 network motifs were suggested to be recurring circuit elements that carry out key information processing tasks. The three-node motif included 13 types of connected subgraphs, such as V-out, 3-Chain, feed forward loop (FFL), 3-Loop, and Clique. Among them, the FFL motif consists of two input regulators, A and B, and one output factor C, where regulators A and B regulate target factor C together, and A also regulates B, as shown in Figure 1. According to the regulation functions (activate or inhibit) between the three elements in FFL, it can be divided into two categories: coherent feed-forward loop and incoherent feed-forward loop (Mangan et al., 2003; Alon, 2007). In the coherent feed-forward loop, the regulators strengthen each other’s functions, and have the effect of controlling stability and resisting noise in biological networks (Le and Kwon, 2013). In the incoherent feed-forward loop, the regulator performs the opposite function to speed up the response and suppress the delay (Kim et al., 2008; Lan and Tu, 2013). FFL plays an important role in biological processing (Milo et al., 2002; Mangan and Alon, 2003), which appears in hundreds of gene systems in *Escherichia coli*, yeast, fruit fly, and humans. The FFL motif governs many aspects of normal cell functions, such as creating bistable switches of gene expression in developing tissues for spatial avoidance, controlling the time sequence of gene expression to create temporal avoidance, and minimizing expression fluctuation against noise (Shalgi et al., 2009).

The Tibetan pig and Rongchang pig are two indigenous pig breeds in China. Rongchang pigs are cross-fertile relatives of Tibetan pigs, living in geographically neighboring low-altitude regions (Tang et al., 2017). Ai et al. (2014) found that Tibetan pigs had a close genetic distance with Rongchang pigs through neighbor-joining (NJ) tree analysis. In this study, based on transcriptional data from six tissues in Tibetan pigs and Rongchang pigs, the key module of lung tissues was identified by constructing a gene network. By integrating complex network analysis and bioinformatics analysis, we identified key genes and size-3 network motifs and found that *KLF4*, a key regulator of the complex, may enhance the survival ability of Tibetan pigs by mediating the TGF-β signaling pathway in the hypoxia environments. This study provides a valuable clue to further understand the molecular mechanism of adaptability in high-altitude hypoxia.

## Materials and Methods

### Gene Expression Data Collection

The protein-encoding genes and miRNA expression profile data of six tissues (muscle, liver, heart, spleen, kidneys, and lungs) from three Tibetan pigs and three Rongchang pigs were obtained from the Gene Expression Omnibus (GEO) database at the National Center for Biotechnology Information (NCBI) under accession numbers GSE93855 (provided by Tang et al., 2017) and GSE124418 (uploaded by Long et al., 2019), respectively. For details about the experimental animals, please refer to the Supplementary Material. Gene list, expressed in each tissue, was updated based on the Sus scrofa 11.1 genome assembly. Taking into account that genes are with very low expression and are less reliable and indistinguishable from the sampling noise, we selected the top 50% of protein-encoding genes of the median absolute deviation (MAD) of expression level.

### Co-expression Network Analysis

Network analysis was performed according to the protocol of the WGCNA R package (Langfelder and Horvath, 2008). We used the following criteria to identify the key module of each tissue: (1) the *p*-value of the correlation between the module and the tissue was less than 3.97×10^−4^ (0.05/126) using the Bonferroni correction method, and (2) the median of the gene significance (GS) value was greater than 0.8. In addition, we calculated the fundamental topology concepts of each key module, including density, mean cluster coefficient, centralization, and heterogeneity.

### Analysis of Gene Expression Patterns in Multiple Tissues

In this study, we used the Mfuzz package in R (Kumar and Futschik, 2007) to identify multitissue expression patterns of each gene in each key module. Based on the fuzzy c-means algorithm, this software implements soft clustering methods for microarray data analysis, which makes the clustering process less sensitive to noise and effectively reflects the strength of a gene’s association with a given cluster.

### Gene Tissue-Specific Analysis

We used the tissue-specificity index (TSI, τ) (Yanai et al., 2005) to grade the scalar measure of the specificity of an expression profile, which ranged from 0 for housekeeping genes to 1 for strictly TS genes. According to Yanai et al. (2005), genes with TSI > 0.9 were considered TS genes.

### Functional Enrichment Analysis of Genes in Key Modules

We used the online software DAVID (v6.8) (Huang et al., 2009) to perform functional enrichment analysis of genes in each key module with all protein-encoding genes on pig genome as the background genes set, including gene ontology (GO) and KEGG pathway analysis.

### Identification of Hub Genes in Key Modules

We identified the hub genes in each key module according to the following criteria: (1) GS value of the gene ≥ 0.8, (2) module membership (MM) value of the gene ≥ 0.95, and (3) in each module, Kwithin ranked in the top 20% of the genes. The module membership (MM) is defined as the correlation of the module eigengenes and the gene expression profile. The Kwithin value represents the degree of connectivity of edges located under the same module as the gene.

### Gene Regulatory Network Construction

We used the TFBSTools package in R (Tan and Lenhard, 2016) to predict the target genes of TFs in each key module of Tibetan pigs. The relScore value was set to 0.85, and other parameters were defaulted. Based on the miRanda tool (Enright et al., 2003), we predicted target genes of the miRNAs, and the Tot Score and Tot Energy were set to 140 and −20, respectively. The gene regulatory network in each Tibetan pig tissue was constructed by combining TFs, miRNAs, target genes, co-expressed genes, hub genes, and their interactions.

### Motif Analysis of the Gene Regulatory Network

The three-node motifs in the gene regulatory network of each tissue were obtained using mfinder1.2 (Kashtan et al., 2004). The number of random networks was set to 10,000. The Z score describes the significance of the difference between the frequency of motifs in the real network and that in the corresponding randomized network. The significance profile (SP) is the vector of Z scores normalized to length 1, describing the statistical significance of each motif in the network (Milo et al., 2004). We constructed the triad significance profile (TSP) of the six tissues from Tibetan pigs, which display certain relations between subgraph types.

### Identification of Important Genes and Size-3 Subgraphs in the Lung-Specific Gene Regulation Network

We calculated the importance scores of each node, edge, and size-3 sub-graph in the lung-specific gene regulatory network. Each node was scored according to the connectivity, differential expression between different conditions, tissue-specific expression, and TF characteristics. The score of each edge was the weight value of the edge from WGCNA. The score of each candidate size-3 sub-graph was calculated by combining the node score and the edge score. The calculation formula and specific details are referred to the Supplementary Material.

### Verification of Important Genes in Lung Tissue

The lung tissue expression profiles of three Tibetan sheep and three yaks were obtained from the GEO database (accession: GSE93855) (Tang et al., 2017), and the expression profiles in the lung tissue of four Diqing Tibetan pigs were collected from another dataset (accession: GSE84409) (Jia et al., 2016). We used WGCNA to perform module analysis. The Hmisc package in R was used to statistically test the correlation between genes.

## Results

### WGCNA and Identification of Key Modules in Tissues

**FIGURE 2:**
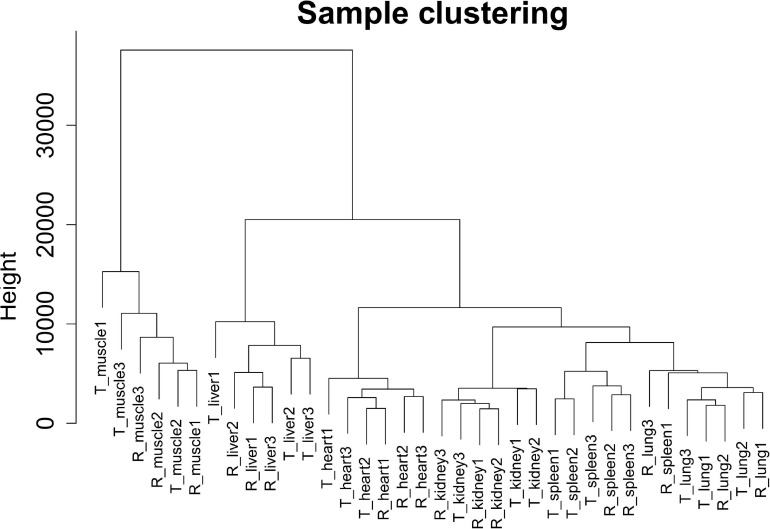
Clustering dendrogram of 36 tissue samples of Tibetan pigs and Rongchang pigs. The figure shows the clustering of a total of 36 tissue samples of Tibetan pigs and Rongchang pigs, where “T” represents Tibetan pigs, and “R” represents Rongchang pigs. For example, “T_muscle1” represents the muscle sample of the first individual Tibetan pig.

In this study, we selected 5,723 protein-coding genes (top 50% of the MAD value) for subsequent analysis. Cluster analysis revealed that different samples from the same tissue of Tibetan pigs and Rongchang pigs clustered together, and no outlier samples were observed, as shown in Figure 2.

**FIGURE 3:**
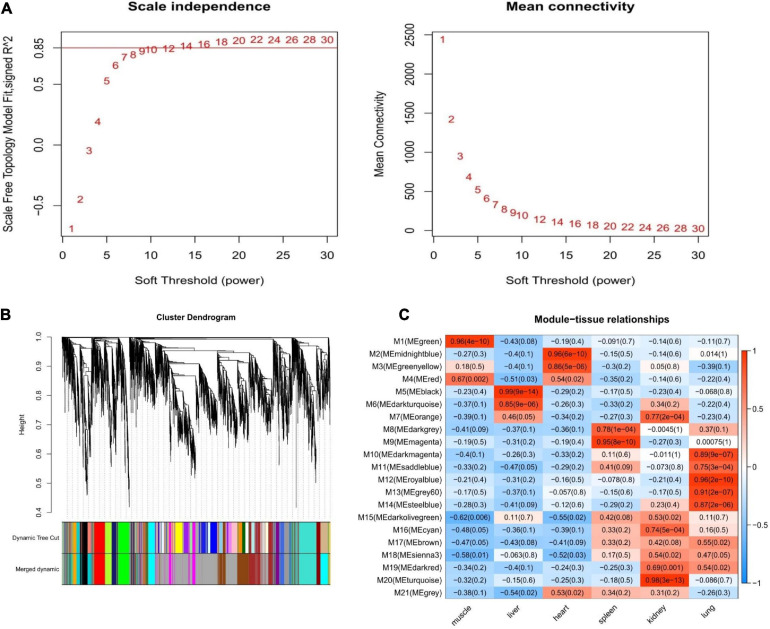
Weighted gene co-expression network analysis of Tibetan pigs. **(A)** Network topology of different soft-thresholding power of Tibetan pig co-expression network. The left panel displays the influence of soft-thresholding power (*x*-axis) on scale-free fit index (*y*-axis). The right panel shows the influence of soft-thresholding power (*x*-axis) on the mean connectivity (degree, *y*-axis). **(B)** Gene clustering module of Tibetan pig co-expression network. The dissimilarity was based on topological overlap. The “Merged dynamic” is the result of merging modules with a correlation higher than 0.9. The *y*-axis is the distance determined by the extent of topological overlap. **(C)** Heatmap of the correlation between module eigengenes and the six tissues of Tibetan pigs. The *x*-axis is the six tissues of Tibetan pigs, and the *y*-axis is the module eigengene (ME). In the heatmap, red represents high adjacency (positive correlation), and blue represents low adjacency (negative correlation). In brackets is the *p*-value of the correlation test.

We constructed a co-expression network for Tibetan pigs and Rongchang pigs. To fulfill the criteria of approximate scale-free topology, the soft threshold power β was set to 20 [the scale-free topological index R^2^ = 0.85 for Tibetan pigs (Figure 3A) and R^2^ = 0.80 for Rongchang pigs (Supplementary Figure 1A)]. Through hierarchic clustering and dynamic branch cutting procedures, 21 and 20 merged modules were identified in the co-expression network of Tibetan pigs and Rongchang pigs, respectively. Clustering of the modules is shown in Figure 3B.

Next, the GS values of genes contained in these modules and the correlation between each module and different tissues in Tibetan pigs (Figure 3C) were calculated. According to the screening criteria, key modules from six tissues (muscle, liver, heart, spleen, kidneys, and lungs) in Tibetan pigs were determined. These modules contained 267, 215, 157, 201, 420, and 350 genes, respectively. The list of genes and the co-expression network for each tissue are shown in Supplementary Table 1 and Supplementary Figure 2, respectively. The gene list of each key module of Rongchang pigs are shown in Supplementary Table 1.

### Network Topology Analysis

We calculated the network topology of the key module of each tissue from the Tibetan pigs and Rongchang pigs, including density, mean cluster coefficient, centralization, and heterogeneity. The results are shown in Table 1. We observed that the network density and the clustering coefficient of Tibetan pig lung and heart tissues were the lowest, while those of the spleen were the highest. These network concepts indicated that the key modules of the lungs and heart were a sparse network. The network topology of Rongchang pigs was similar to those of Tibetan pigs.

**TABLE 1:** The fundamental network topology concepts of key modules in Tibetan pig and Rongchang pig tissues.

**Pig breed**	**Tissue**	**Key module**	**DS**	**MCC**	**CL**	**HG**
Tibetan pig	Muscle	M1	0.03	0.26	0.10	1.12
	Liver	M5	0.05	0.21	0.13	1.10
	Heart	M2	0.03	0.14	0.09	0.91
	Spleen	M9	0.12	0.28	0.17	0.80
	Kidney	M20	0.05	0.17	0.11	0.84
	Lung	M22	0.03	0.13	0.08	0.82
Rongchang	Muscle	M14	0.05	0.18	0.12	1.01
	Liver	M8	0.05	0.20	0.13	1.08
	Heart	M21	0.05	0.18	0.11	0.97
	Spleen	M3	0.08	0.22	0.14	0.81
	Kidney	M13	0.07	0.20	0.14	0.83
	Lung	M1	0.05	0.17	0.10	0.76

*DS, network density; MCC, mean cluster coefficient; CL, network centralization; Hg, network heterogeneity.*

### Multitissue Gene Expression Patterns

**FIGURE 4:**
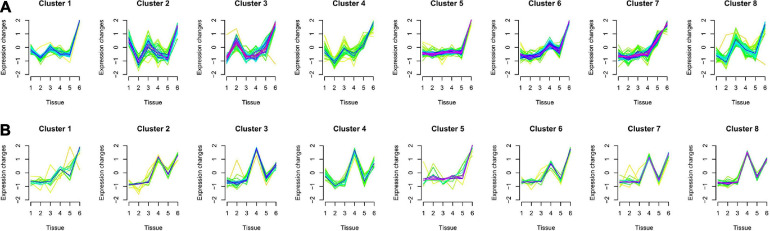
Multitissue expression patterns of genes in key modules of lung tissue of two pig breeds. **(A)** Multitissue expression patterns of key module genes in Tibetan pig lung tissues. **(B)** Multitissue expression patterns of key module genes in Rongchang pig lung tissues. The 1, 2, 3, 4, 5, and 6 in the figure represent the muscle, liver, heart, spleen, kidneys, and lung tissues, respectively. The yellow- or green-colored lines correspond to genes with low membership value; the red- and purple-colored lines correspond to genes with high membership value.

According to the analysis of gene expression patterns, we found that compared with other tissues, Tibetan pigs and Rongchang pigs had the largest differences in gene expression patterns of the key modules of lung tissue. In the key module of lung tissue, gene expression patterns in multitissues could be divided into eight clusters. Compared with other tissues, the level of gene expression in the lung tissues of Tibetan pigs was the highest in all clusters (Figure 4A). However, the genes in the lung tissues of Rongchang pigs are expressed as the highest only in cluster 1, cluster 5, and cluster 6 (Figure 4B). The different gene expression patterns may be caused by physiological changes in the hypoxia environment of Tibetan pigs or interspecies differences between Tibetan pigs and Rongchang pigs.

### Tissue-Specific Gene Analysis

A total of 210 and 206 genes were identified as tissue specific (τ > 0.9) in the key modules of Tibetan pigs and Rongchang pigs, respectively. For the gene details, see Supplementary Table 2. For Tibetan pigs, there were 32, 50, 23, 36, 47, and 22 TS genes in the key modules of the muscle, liver, heart, spleen, kidneys and lungs, respectively. There are more TS genes in the lung tissues of Tibetan pigs than in Rongchang pigs. Compared with the other five tissues, the number of TS genes in the lung has the largest difference between the two pig breeds.

### Functional Enrichment Analysis of Genes in Key Modules

To further understand the biological functions of genes in each key module in Tibetan pigs and Rongchang pigs, we conducted gene function enrichment analysis. After the Benjamini correction, we identified significant pathway enrichment in Tibetan pigs and Rongchang pigs, as shown in Supplementary Table 3. Compared with Rongchang pigs, there were 10, 4, 1, and 13 pathways in the muscle, lungs, heart, and spleen that were only significantly enriched in Tibetan pigs, as shown in Table 2. Pathways enriched only in Tibetan pig lungs, including regulated cell growth, proliferation, migration, and apoptosis include focal adhesion (ssc04510), ECM–receptor interaction (ssc04512), PI3K–Akt signaling pathway (ssc04151), and TGF-β signaling pathway (ssc04350). In addition, 30 pathways were significantly enriched only in Rongchang pigs (see Supplementary Table 4).

**TABLE 2:** Pathways that are only significantly enriched in Tibetan pig tissue modules.

**Tissue**	**Category**	**ID**	**Term**	**Benjamini**
Muscle	Cellular components	GO:0031595	Nuclear proteasome complex	3.97E-03
		GO:0008540	Proteasome regulatory particle, base subcomplex	1.02E-02
	KEGG_Pathway	ssc03050	Proteasome	3.15E-04
		ssc01200	Carbon metabolism	6.64E-03
		ssc04152	AMPK signaling pathway	1.38E-02
		ssc04261	Adrenergic signaling in cardiomyocytes	2.18E-02
		ssc04931	Insulin resistance	3.64E-02
		ssc05169	Epstein–Barr virus infection	3.69E-02
		ssc04722	Neurotrophin signaling pathway	3.83E-02
		ssc04921	Oxytocin signaling pathway	4.11E-02
Heart	KEGG_Pathway	ssc05412	Arrhythmogenic right ventricular cardiomyopathy (ARVC)	1.85E-02
Spleen	Biological progresses	GO:0006412	Translation	6.86E-15
		GO:0001731	Formation of translation preinitiation complex	1.32E-02
		GO:0006446	Regulation of translational initiation	1.81E-02
	Cellular components	GO:0022627	Cytosolic small ribosomal subunit	6.49E-17
		GO:0022625	Cytosolic large ribosomal subunit	8.99E-08
		GO:0016282	Eukaryotic 43S preinitiation complex	1.29E-04
		GO:0033290	Eukaryotic 48S preinitiation complex	1.76E-04
		GO:0005852	Eukaryotic translation initiation factor 3 complex	2.17E-03
		GO:0005683	U7 snRNP	2.35E-02
		GO:0042105	Alpha-beta T-cell receptor complex	2.35E-02
	Molecular function	GO:0003735	Structural constituent of ribosome	1.59E-18
		GO:0003743	Translation initiation factor activity	9.59E-03
	KEGG_Pathway	ssc03010	Ribosome	4.10E-19
Lung	KEGG_Pathway	ssc04510	Focal adhesion	3.69E-05
		ssc04512	ECM–receptor interaction	2.98E-04
		ssc04151	PI3K–Akt signaling pathway	9.26E-03
		ssc04350	TGF-beta signaling pathway	1.80E-02

### Identification of Hub Genes in Key Modules

According to the screening criteria of hub genes, we have determined the hub genes of each key module of Tibetan pigs and Rongchang pigs. Compared with Rongchang pigs, Tibetan pigs had more hub genes in the liver, kidneys, and lung tissues. There was no hub gene overlap between the lung tissues of Tibetan pigs and Rongchang pigs. In addition, eight hub genes were TS gene in the lung tissue of Tibetan pigs, while Rongchang pigs only have one. Table 3 summarizes the hub gene information in Tibetan pigs and Rongchang pigs.

**TABLE 3:** Hub gene information of key modules in Tibetan pigs and Rongchang pigs.

**Pig breed**	**Tissue**	**Num of hubs**	Num of overlapping hubs*	**Num of hub (TSI > 0.9)**	**Num of TFs in hub**
Tibetan pig	Muscle	23	22	11	1
	Liver	41	20	30	0
	Heart	20	2	8	1
	Spleen	40	6	20	1
	Kidney	81	45	26	1
	Lung	32	0	8	6
Rongchang	Muscle	61	22	25	7
	Liver	39	20	30	0
	Heart	26	2	3	0
	Spleen	123	6	0	13
	Kidney	68	45	26	0
	Lung	14	0	1	0

*“Num of overlapping hubs” is the number of overlapping hub genes detected by Tibetan pigs and Rongchang pigs in the same tissue module.

### Gene Regulatory Network Construction

**FIGURE 5:**
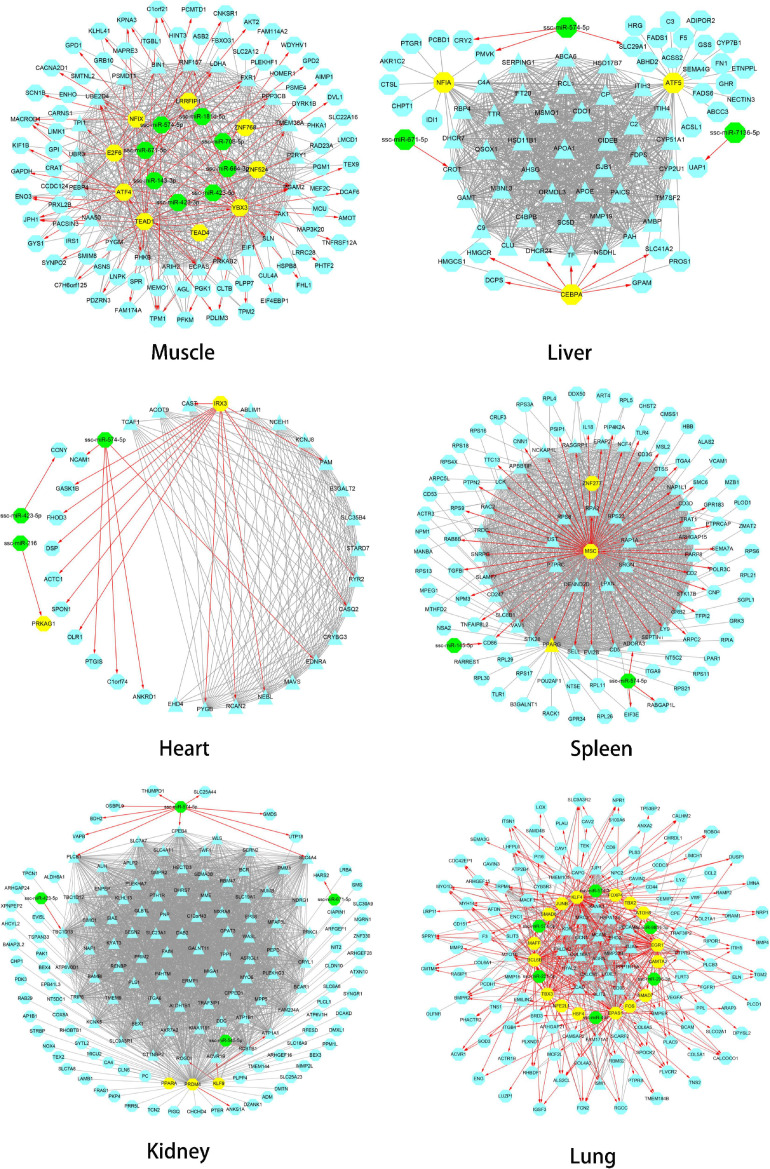
Gene regulatory network of six tissues of Tibetan pigs. In each network in the figure, the yellow dots represent TFs, the green dots represent miRNAs, and the hub genes are represented by triangles. The red edges with arrows represent the regulatory relationship between TFs and miRNAs and target genes. The gray edge indicates that there is only a co-expression relationship between the two genes.

The gene regulatory network of Tibetan pig tissues was constructed by combining TFs, miRNAs, target genes, co-expression genes, and hub genes. There were 115, 80, 35, 117, 160, and 157 nodes (genes) and 986, 1,786, 298, 1,976, 5,315, and 1,075 edges (regulatory relationship) in the gene regulatory network of the muscle, liver, heart, spleen, kidneys, and lungs, respectively, as shown in Figure 5. There were 9, 3, 1, 3, 3, and 16 TFs, respectively, in the gene regulatory network of each tissue. In total, 35 TFs belonged to 10 TF families, among which 10 TFs were also hub genes. According to the PWM provided by the CIS-BP database, 20 TFs target genes were predicted. We found that these 20 TFs regulate 237 genes (94 genes are hub genes) in each tissue key modules, predicting a total of 408 regulatory relationships. Through the prediction of miRNA target genes, we found that genes in the key modules of the muscle, liver, heart, spleen, kidneys, and lungs were regulated by 8, 3, 3, 2, 4, and 6 miRNAs, respectively. Table 4 summarizes the information of TFs, miRNAs, target genes, and hub genes in the gene regulatory network of six tissues in Tibetan pigs.

**TABLE 4:** Detailed information of gene regulatory networks in six tissues of Tibetan pigs.

**Tissue**	**Num of TFs**	**Num of TF target genes**	**Num of hub regulated by TFs**	**Num of miRNAs**	**Num of miRNAs target genes**	**Num of hub regulated by miRNAs**
Muscle	9	49	17	8	12	1
Liver	3	7	3	3	5	1
Heart	1	13	7	3	8	1
Spleen	3	55	33	2	4	1
Kidney	3	4	3	4	13	5
Lung	16	118	31	6	12	3

### Identification of Gene Regulatory Network Motifs

In gene networks, some motifs displayed much higher frequencies than expected in randomized networks (Ravasz et al., 2002; Shen-Orr et al., 2002), and these motifs were suggested to be recurring circuit elements that perform key information-processing tasks (Rosenfeld et al., 2002; Shen-Orr et al., 2002; Mangan and Alon, 2003). Among them, the motif composed of three nodes contains 13 kinds, including V-out, 3-Chain, FFL, 3-Loop, Clique, and so on. Using the mfinder1.2 software, we identified 8,894, 13,067, 993, 19,899, 78,959, and 14,692 motifs in the gene regulatory networks of the muscle, liver, heart, spleen, kidneys, and lung tissues in Tibetan pigs, respectively. There were significant differences in the distribution of motifs among the different gene regulatory networks (*p* < 2.2E-16). The motif information in the gene regulatory networks of the six tissues is shown in Table 5.

**TABLE 5:** Motif information in regulatory networks of six tissues in Tibetan pigs.

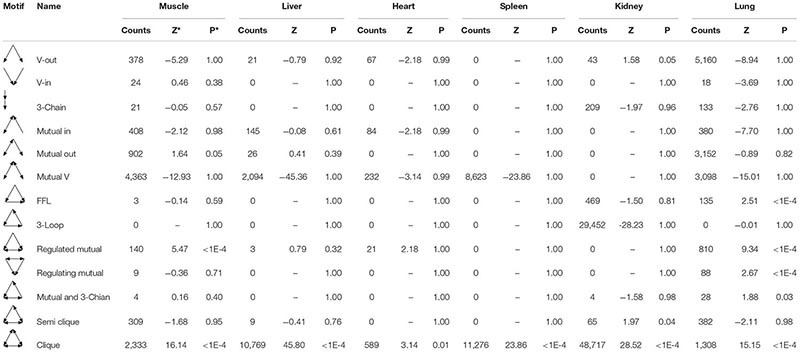

*^∗^“Z” and “P” in the table are the Z score and *p*-value calculated by the mfinder1.2 software for each motif.*

**FIGURE 6:**
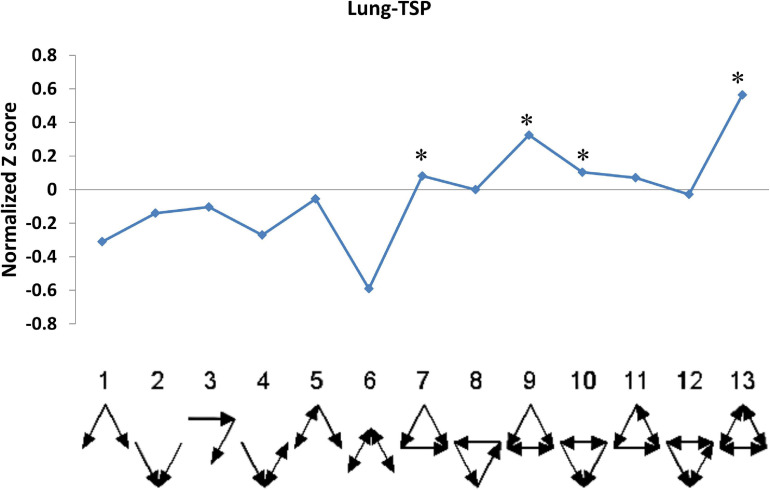
The triad significance profile (TSP) of Tibetan pig lung gene regulatory network. The ordinate in the figure is the normalized Z value, and the abscissa is the 13 motif types. The point marked with “*” is that the frequency of the corresponding motif in the lung tissue gene regulatory network is significantly different from that of random networks (*p* < 1E-04). The motifs are FFL (7), Regulated mutual (9), Regulating mutual (10), and Clique (13) in that order.

To analyze the statistical significance of each motif type, we generated 10,000 random networks representing a conservation rule. The distribution of TSP in the gene regulatory network of the lung tissues is shown in Figure 6. We found that the frequency of FFL, Regulated mutual, Regulating mutual, and Clique motifs in the lung tissue gene regulatory network was significantly different from that of random networks (*p* < 1E-04). In the muscle and heart tissue gene regulatory network, Regulated mutual and Clique motif were significant motif types, while V-out, Semi clique, and Clique motif were significant in the gene regulatory network of the kidneys.

### Motif Analysis of the Gene Regulatory Network in the Lung Tissues

We further analyzed FFL, Regulated mutual, and Regulated mutual motifs in the gene regulatory network of lung tissues. All FFL motifs in the gene regulatory network of lung tissues were TF_1_→TF_2_, including *KLF4→EPAS1*, *KLF4→BCL6B*, *KLF4→FOS*, *EGR1→BCL6B*, *EGR1→EPAS1*, *BCL6B→EPAS1*, *TBX3→EPAS1*, and *TBX3→BCL6B*. Then, the two TFs shared a target gene. As a result, 51 target genes were regulated, including four TFs, forming 13 FFLs, and 21 hub genes, forming 71 FFLs. In addition, three of these target genes were both TF and hub gene, forming eight FFLs.

There were two main types of Regulating mutual motifs. One includes two TFs regulating each other, including *EGR1–KLF4*, *EGR1–TBX3*, and *KLF4–TBX3*, and jointly regulating the same target gene. A total of 47 target genes were regulated, including six TFs, forming 12 complexes, and 27 hub genes, forming 49 complexes. Among these target genes, four target genes were both TF and hub genes, forming 10 complexes. The other type of Regulating mutual motifs includes two TFs that were co-expressed and shared a target gene. We found that *FOS* and *JUNB* co-expressed and co-regulated the *DUSP1* gene.

In the Regulated mutual motif, one TF regulated two genes, and there was a co-expression relationship between the two target genes. It was composed of TFs, including *EGR1*, *KLF4*, *EPAS1*, *BCL6B*, and *TBX3*, and their regulated target genes, forming a total of 810 complexes. Of these complexes, there were eight in which both target genes are TFs and 593 in which both are hub genes. In the Clique motif, only the *EGR1–KLF4–TBX3* motif was the mutual regulation of these three TFs, and the remaining motifs were co-expressed relationships among genes.

### Identification of Important Genes and Regulatory Relationships Related to Hypoxia in the Lung Gene Regulatory Network

Formulas (8) and (10) were used to evaluate the importance of each gene and the three-node motif, including FFL, Regulating mutual, and Regulated mutual type motif, in the gene regulatory network of lung tissues. We found that the several important top genes were *KLF4*, *BCL6B*, *EGR1*, *SMAD6*, and *EPAS1* transcription factor genes, which were also hub genes. The top 25% of the node importance scores in the Tibetan pig lung gene regulatory network are shown in Table 6.

**TABLE 6:** The top 25% of S_node_ genes in the Tibetan pig lung gene regulatory network.

**Ranking**	**Gene**	**Full name**	Type (Hub/TF)*	**S_*node*_**
1	*KLF4*	Kruppel-like factor 4	Hub & TF	0.4485
2	*BCL6B*	BCL6B transcription repressor	Hub & TF	0.3506
3	*EGR1*	Early growth response 1	Hub & TF	0.3184
4	*TBX3*	T-box 3	TF	0.1885
5	*EPAS1*	Endothelial PAS domain protein 1	Hub & TF	0.1789
6	*SMAD6*	SMAD family member 6	Hub & TF	0.1516
7	*MAFF*	MAF bZIP transcription factor F	TF	0.1189
8	*CCN1*	Cellular communication network factor 1	Hub	0.0814
9	*GJA5*	Gap junction protein, alpha 5	Hub	0.0784
10	*JUNB*	JunB proto-oncogene, AP-1 transcription factor subunit	TF	0.0744
11	*CALCRL*	Calcitonin receptor like receptor	Hub	0.0712
12	*FOS*	Fos proto-oncogene, AP-1 transcription factor subunit	TF	0.0707
13	*JCAD*	Junctional cadherin 5 associated	Hub	0.0705
14	*MRC2*	Mannose receptor C type 2	Hub	0.0683
15	*PECAM1*	Platelet and endothelial cell adhesion molecule 1	Hub	0.0646
16	*TJP1*	Tight junction protein 1	Hub	0.0645
17	*CD93*	CD93 molecule	Hub	0.0636
18	*LHFPL6*	LHFPL tetraspan subfamily member 6	TG	0.0611
19	*COL16A1*	Collagen, type XVI, alpha 1	Hub	0.0588
20	*PTPRB*	Protein tyrosine phosphatase receptor type B	Hub	0.0570
21	*SMAD7*	SMAD family member 7	Hub	0.0556
22	*MCAM*	Melanoma cell adhesion molecule	Hub	0.0540
23	*HYAL2*	Hyaluronidase 2	Hub	0.0525
24	*SLIT2*	Slit guidance ligand 2	Hub	0.0515
25	*HSPA12B*	Heat shock protein family A (Hsp70) member 12B	Hub	0.0495
26	*PPP1R15A*	Protein phosphatase 1, regulatory subunit 15A	Hub	0.0464
27	*EHD2*	EH domain containing 2	Hub	0.0461
28	*PHLDA2*	Pleckstrin homology like domain family A member 2	Hub	0.0451
29	*KANK3*	KN motif and ankyrin repeat domains 3	Hub	0.0442
30	*MMP23B*	Matrix metallopeptidase 23B	Hub	0.0394
31	*LOXL1*	Lysyl oxidase like 1	Hub	0.0333
32	*TBX2*	T-box 2	TF	0.0251
33	*FAM171A1*	Family with sequence similarity 171 member A1	Hub	0.0250
34	*KDR*	Kinase insert domain receptor	Hub	0.0212
35	*MYO1C*	Myosin IC	Hub	0.0211
36	*ATOH8*	Atonal bHLH transcription factor 8	TF	0.0193
37	*AGRN*	Agrin	Hub	0.0178
38	*NPC2*	NPC intracellular cholesterol transporter 2	TG	0.0169
39	*PLAC9*	Placenta associated 9	TG	0.0163
40	*SLC9A3R2*	SLC9A3 regulator 2	TG	0.0157

*There are four types of genes, including “Hub&TF,” “Hub,” “TF,” and “TG.” Among them, type “Hub & TF” represents that the gene is both TF and hub genes. Type “Hub” represents that the gene is a hub gene, and type “TF” represents that the gene is TF. Type “TG” means that the gene is neither hub nor TF, but only a target gene of TF.

**FIGURE 7:**
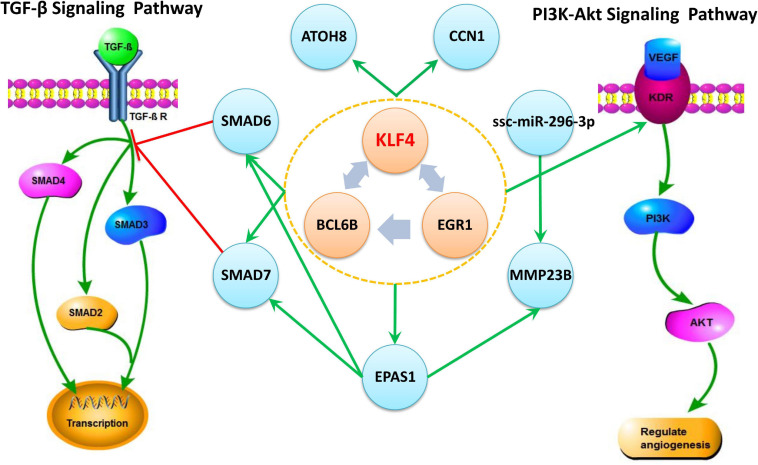
The *“KLF4–EGR1–BCL6B”* complex and their regulated genes in Tibetan pig lung tissues. The complex formed by *KLF4–EGR1–BCL6B* regulates the *EPAS1*, *SMAD6*, *SMAD7*, *KDR*, *ATOH8*, and *CCN1* genes and mediates the TGF-β and PI3K-Akt signaling pathways by regulating *SMAD6*, *SMAD7*, and *KDR* genes, respectively. The green edge in the figure represents regulation, and the red edge represents inhibition.

The Regulating mutual motif formed by *KLF4–EGR1–BCL6B* was the most important motif based on the motif score. We call it the “*KLF4–EGR1–BCL6B*” complex. In this motif, the *KLF4* and *EGR1* genes regulate the same target gene, *BCL6B*. This complex preferred to synergistically regulate the *EPAS1*, *KDR*, *SMAD6*, *SMAD7*, *CCN1*, and *ATOH8* genes (Figure 7), which comprised 18 motifs (Table 7). The “*KLF4–EGR1–BCL6B*” complex may coordinately regulate the *SMAD6* and *SMAD7* genes, which play an important role in the TGF-β signaling pathway. *EPAS1* is an important hypoxia-inducible factor. This complex may also indirectly regulate *SMAD6* and *SMAD7* genes by regulating the *EPAS1* gene. This complex may also regulate the *KDR* gene, which is involved in the PI3K–Akt signaling pathway. Both TGF-β and PI3K–Akt signaling pathways play an important role in hypoxia response (Chen et al., 2006; Ambalavanan et al., 2008; Jia et al., 2016; Qi et al., 2019).

**TABLE 7:** The motifs formed between the “*KLF4–EGR1–BCL6B*” complex and its regulatory genes in the lung.

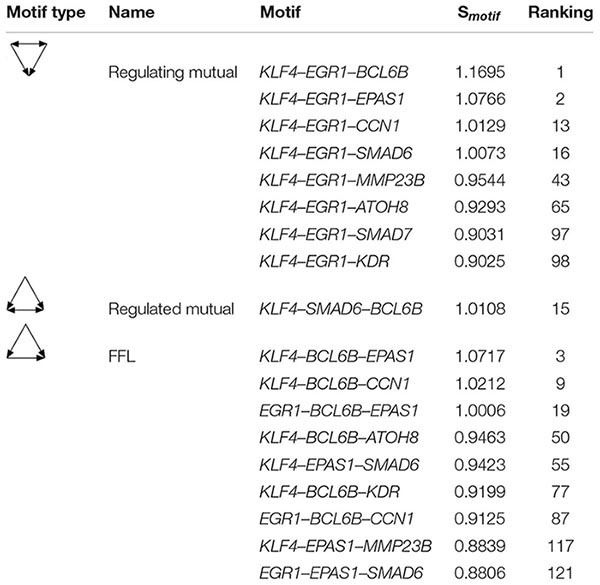

### Validation of Important Genes in Lung Tissue

To confirm the relationship between *KLF4*, *EGR1*, *BCL6B*, *SMAD6*, *EPAS1*, *KDR*, *SMAD7*, *CCN1*, *ATOH8*, and *MMP23B* genes, we used lung tissue transcriptomic data from the Tibetan sheep, yak, and Diqing Tibetan pig population for validation via the co-expression network analysis. We identified the key module of the lung tissues for the three validation groups using WGCNA. The network topology of these key modules, including density, mean cluster coefficient, centralization, and heterogeneity, were similar to those of Songpan Tibetan pigs. There were 151, 150, and 73 overlap genes between gene sets of the lung key module for the three validation groups and Tibetan pigs, respectively.

**FIGURE 8:**
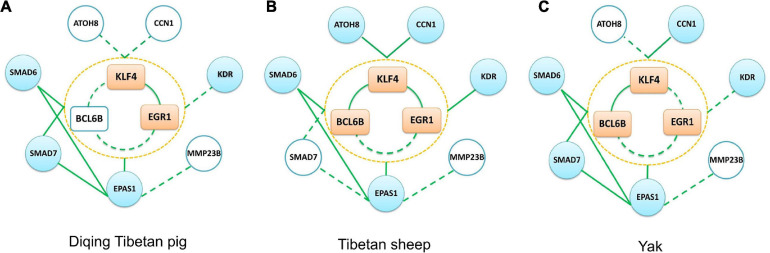
Three other groups (Diqing Tibetan pig, Tibetan sheep, and yak) verification network. **(A–C)** The verification results of the Diqing Tibetan pig, Tibetan sheep, and yak, respectively. The peach-colored rectangle in the middle part of each subgraph represents the *KLF4–EGR1–BCL6B* complex, the sky blue circle represents the target gene, and the white circle represents that this gene is not included in the data set. The solid green line in the figure represents the edge we verified, and the dotted line represents the unverified edge.

Due to using commercially available Agilent Whole Porcine Genome Oligo (4 × 44 K) Microarrays for Diqing Tibetan pig, there was no probe annotation information for the *BCL6B*, *CCN1*, *ATOH8*, and *MMP23B* genes. In Diqing Tibetan pig lung tissues, six genes, including *KLF4*, *EGR1*, *EPAS1*, *SMAD6*, *SMAD7*, and *KDR*, were highly expressed and were significantly positively correlated between genes, except for between *KDR* gene and others (as shown in the Supplementary Table 5). Overall, there were 11 and nine edges (the co-expression relationship) among the above genes found in the Songpan Tibetan pig and Diqing Tibetan pig (Figure 8A), respectively. So, 81.82% (9/11) of the co-expression relationships in the above genes were confirmed. Based on the WGCNA for the Diqing Tibetan pig lung tissue, we found the *KLF4*, *EGR1*, and *SMAD7* genes were clustered into one module, while *EPAS1* and *SMAD6* were clustered into the other module.

The *KLF4*, *BCL6B*, *EPAS1*, *EGR1*, *SMAD6*, *KDR*, *CCN1*, and *ATOH8* genes had the highest expression in lung tissues compared with the other five tissues of Tibetan sheep (muscle, liver, heart, spleen, and kidneys). There were 14 and 24 significantly co-expression relationships among the above genes, which were identified in Tibetan sheep (see Figure 8B and the Supplementary Table 5) and Songpan Tibetan pig, respectively. In total, 58.33% (14/24) of the relationships between genes were validated. Except for *EGR1* and *ATOH8*, the other genes were all clustered into the same key module related to the lung using WGCNA.

With the exception of *ATOH8* and *MMP23B*, the other genes were most highly expressed in the lung tissues of yak compared with the other five tissues. We detected 12 and 24 significantly positive correlations in the above genes of Tibetan yak lung tissue (see Figure 8C and the Supplementary Table 5) and Songpan Tibetan pig. We successfully verified 50% (12/24) of the relationships among genes. After performing WGCNA, we found that *KLF4*, *BCL6B*, *EPAS1*, *SMAD6*, and *SMAD7* were clustered into key modules related to the yak lungs.

## Discussion

Many previous studies primarily focused on identifying differentially expressed genes through gene expression profile analysis, but interactions between genes in different cell states may not have been fully considered (Kostka and Spang, 2004). Moreover, differences in gene expression are not equal to differences in gene action. Compared with expression level analysis, network-based analysis not only captures local patterns but also identifies global patterns in a biological context, revealing molecular regulation details of hub genes at the network level. At the same time, the hub genes related to biological processes identified through gene network analysis also provide clues for subsequent molecular studies.

In this study, we detected the gene regulatory network related to Tibetan pig lung tissues. An appropriate sample size is critical to the planning and interpretation of genetic studies, whether they are descriptive or analytical. The small sample sizes will result in imprecise estimates in a descriptive study and failure to achieve statistical significance in an analytic or comparative study (Weber and Hoo, 2018). Due to the sample size limitation of this study, this may have limited the generalizability of the results of the research, and further independent tests may be required to verify our findings. However, our research methods and results can provide some valuable clues for the study of the hypoxia adaptation mechanism of Tibetan pigs. Combining topological characteristics, differential expression, and tissue-specific expression, we identified a list of genes that may be related to hypoxia in Tibetan pig lung tissue, such as *EPAS1*, *LOXL1*, *KLF4*, *EGR1*, *BCL6B*, *SMAD6*, *SMAD7*, *KDR*, *MMP23B*, and miR-296.

The *EPAS1* gene found in this study may be related to the adaptation to the hypoxic environment. The *EPAS1* gene encodes one subunit of hypoxia-inducible factor (HIF), which show multifarious effects involved in complex oxygen sensing (Henderson et al., 2005) and regulation of angiogenesis, hemoglobin concentration, and erythrocytosis (Beall et al., 2010). In the Tibetan human population, the *EPAS1* gene is involved in the chronic hypoxia response, and it has been shown to have a strong signature of selection (Bigham et al., 2010; Simonson et al., 2010; Peng et al., 2011; Xu et al., 2011). Moreover, Li et al. (2019) show that the mutant genotype frequencies of the rs13419896, rs1868092, and rs4953354 loci in the *EPAS1* gene are significantly higher in the Tibetan population than in the plains population. Under plateau hypoxic conditions, the plains population was able to acclimate rapidly to hypoxia through increasing *EPAS1* mRNA expression and changing the hemoglobin conformation. The *EPAS1* gene also has obvious selection signature in other plateau animals, such as Tibetan horses (Liu et al., 2019) and Tibetan pigs (Ma et al., 2019) and has been identified as a key evolutionary molecule adapted to the plateau hypoxic environment.

Angiogenesis was an adaptive response to tissue hypoxia (Fong, 2008). A majority of the identified hub genes participated in the angiogenesis process, such as *LOXL1*, *KLF4*, and *EGR1*. The *LOXL1* gene is essential for the stability and strength of elastic vessels and tissues (Li et al., 2020) and may play important roles in the enhanced angiogenesis promoted by hypoxia (Xie et al., 2017). The *KLF4* gene tended to be pleiotropic. Not only does it promote pulmonary angiogenesis and blood transport (Ghaleb and Yang, 2017) and accelerate the acquisition and transport of oxygen but also it protects the lungs from hypoxia (Shatat et al., 2014). The *EGR1* gene stimulates and induces the process of angiogenesis (Adamson and Mercola, 2002; Sheng et al., 2018). Angiogenesis is conducive to the increase in oxygen transport. Therefore, we infer that these genes might contribute to obtaining and transporting oxygen better in hypoxic environments, by involving in the angiogenesis process.

Based on the Tibetan pig lung tissue-specific gene network, we found that *KLF4* and *EGR1* simultaneously regulated the *BCL6B* gene, forming the *KLF4–EGR1–BCL6B* complex, which might be dominated by the *KLF4* gene and affect the expression of *EPAS1*, *SMAD6*, *SMAD7*, *CCN1*, *KDR*, and *ATOH8*. These key genes and regulatory relationships were validated in the lung tissues of Tibetan pigs from Jia et al. (2016) and Tibetan sheep and yak from Tang et al. (2017). After a large literature review and verification of gene function annotation, we postulate that the *KLF4–EGR1–BCL6B* complex might be beneficial for Tibetan pigs to survive better in hypoxic environments.

The *KLF4*, *EGR1*, and *BCL6B* genes jointly regulate the *SMAD6* and *SMAD7* genes, which are important regulators of the TGF-β signaling pathway. In the TGF-β signaling pathway, the SMAD family genes are very important signal transduction and regulatory molecules. *SMAD6* and *SMAD7* are antagonists of the TGF-β gene family. High expression of *SMAD7* inhibited the transcription of *SMAD2* and *SMAD3* gene induced by the *TGF-β* gene and antagonizes tissue fibrosis (Yan et al., 2009). Therefore, the *KLF4–EGR1–BCL6B* complex in Tibetan pig lungs may mediate the TGF-β signaling pathway by regulating the expression of *SMAD6* and *SMAD7*, thereby enhancing the antifibrotic effect of the lungs.

Moreover, the *KLF4–EGR1–BCL6B* complex might regulate the *KDR* gene, which was primarily expressed in pulmonary vascular endothelial cells and has important proangiogenic activity (Melincovici et al., 2018). The *KDR* gene is an important regulator of the PI3K–Akt signaling pathway. Jia et al. (2016) and Qi et al. (2019) found that the PI3K–Akt signaling pathway was involved in hypoxia adaptation in both Tibetan pigs and yaks. Under hypoxic conditions, the combination of *KDR* and *VEGF* activates the downstream *PI3K* gene, thereby regulating proliferation and differentiation of neovascular endothelial cells and playing an important role in the development of angiogenesis (Graupera and Potente, 2013). Therefore, the *KLF4–EGR1–BCL6B* complex may act on the PI3K–Akt pathway by mediating the *KDR* gene and accelerating the acquisition and transportation of oxygen under hypoxic conditions.

In addition, the *KLF4–EGR1–BCL6B* complex also regulated the *ATOH8*, *CCN1*, and *EPAS1* genes. High expression of *CCN1* suppressed pulmonary vascular smooth muscle contraction in response to hypoxia (Lee et al., 2015). The *ATOH8* gene participates in the *ALK-1/SMAD/ATOH8* axis, which attenuated the hypoxic response in endothelial cells in the pulmonary circulation and might help prevent the development of pulmonary arterial hypertension (Morikawa et al., 2019). The *MMP23B* gene was a member of the MMP gene family, and MMP matrix metalloproteinases played an important role in tissue remodeling and angiogenesis (Białkowska et al., 2020). Moreover, *MMP23B* is regulated by *EPAS1* and ssc-miR-296-3p. Studies had shown that miR-296 can regulate angiogenesis (Anand and Cheresh, 2011; Li et al., 2018).

The co-expression and network analysis were performed in three validation groups (Tibetan sheep, yak, and Diqing Tibetan pig). Comparing pigs, sheep, and cow living in normal oxygen content environments, some genes, such as *KLF4*, *EGR1*, *EPAS1*, *SMAD6*, and *KDR* genes, are overlapped in the key module of the Tibetan pig, Tibetan sheep, and yak lung tissues. As stated above, these genes improve the tolerance of Tibetan pigs to hypoxic environment through involving in angiogenesis and antagonizing lung tissue fibrosis.

Due to using porcine oligo microarrays for the Diqing Tibetan pig, some genes, such as *BCL6B*, do not have probe annotation information. We did not observe the *KLF4–EGR1–BCL6B* complex in the Diqing Tibetan pig lungs, but the *KLF4* and *EGR1* genes might jointly correlate with *SMAD6*, *SMAD7*, and *EPAS1* genes. In the Tibetan sheep lung, the *BCL6B* gene did not significantly correlate with the *EGR1* gene (*p* value = 0.0839), due to the limitation of sample size. So, further experiments in a large validation population, such as ChIP-seq, would help the demonstration of the regulation function of the complex *KLF4–EGR1–BCL6B*.

Although we identified the *KLF4* gene as a key gene in the lung tissue of different species, and related to the *SAMD6* and *EPAS1* genes, there are some different co-expression relationships in the gene regulatory network of Tibetan pig, Tibetan sheep, and yak. We deem that the following reasons could have contributed to the observed differences. First, the study populations of different species have various genetic backgrounds. Many previous studies have also shown that there are different anatomical structures of tissues, physiological and biochemical indexes, and molecular mechanisms in the environment adaptation of plateau animals. Second, there are differences in the sampling methods and genetic drift events among studies on the same species. Third, gene regulatory programs display a wide range of characteristics, depending on where they are in the body and what stage in its life cycle. To control a cell’s behavior in different space and time, different gene expression profiles and regulation relationship will be observed.

In addition to the lung tissues, the heart also plays an important role in high-altitude hypoxia adaptation. Qi et al. (2019) showed that the heart and lung tissues were identified as the two key organs of yak hypoxia adaptation. In this study, we identified some specific expression genes related to hypoxia in the heart tissue of Songpan Tibetan pigs, such as *EGLN3*, *RYR2*, *EDNRA*, and *EGLN3* (egl-9 family hypoxia inducible factor 3), that likely plays an important role in cellular adaptation to hypoxic conditions by participating in the HIL-1 signaling pathway. Zhang B. et al. (2017) used the transcriptomic and proteomic data of Tibetan pig heart tissues to identify the *EGLN3* gene as important candidate genes for high-altitude adaptation. Moreover, the *EGLN1* gene, a member of the same family of *EGLN3*, has been determined to be involved in the hypoxia adaptation of Tibetan humans (Bigham et al., 2010; Simonson et al., 2010; Xiang et al., 2013).

*RYR2* and *EDNRA* may play crucial roles in heart development, heart rhythm stabilization, and signal transduction to cope with hypoxia. Zhang et al. (2014) found that the *RYR2* gene was related to the hypoxia adaptability of Tibetan gray wolves. Low oxygen environment can increase *EDNRA* gene expression (Minchenko et al., 2019; Zhang Y. et al., 2017). However, Zhang B. et al. (2017) did not identify the *RYR2* and *EDNRA* genes as hypoxia-related candidate genes by comparative mRNA and protein expression profiles in heart tissues of Tibetan and Yorkshine pigs, respectively. These inconsistencies may be a result of differences in the study population in the genetic backgrounds, population structure, developmental stages, and environmental factors. This also suggests that many key genes, in reducing hypoxia injury that exists within the Tibetan pig genome, have yet to be discovered.

## Conclusion

In summary, through gene network analysis, we found that lung tissues may play an important role in hypoxia adaptation in Tibetan pigs. We comprehensively profiled the gene regulatory network of Tibetan pig lung tissues, identifying a series of candidate genes related to hypoxia and discovering that *KLF4* is likely the core regulator of the *KLF4–EGR1–BCL6B* complex, which may mediate the TGF-β signaling pathway and improve the anti-hypoxic ability of Tibetan pigs. Although gene function is not entirely dependent on gene expression regulation, these findings may provide valuable clues and better understanding in exploring the underlying molecular mechanisms of hypoxia adaptation.

## Data Availability Statement

Publicly available datasets were analyzed in this study. This data can be found here: The data underlying this article are available in Gene Expression Omnibus (GEO) database at https://www.ncbi.nlm.nih.gov/geo/, and can be accessed with GSE93855, GSE124418, and GSE84409.

## Author Contributions

ZW, XW, and TW conceived the project. TW, YG, SL, and CZ performed the bioinformatics and data analysis. TW and ZW wrote the manuscript. TC, KD, and PW collected the samples and data. All authors read and approved the final manuscript.

## Conflict of Interest

The authors declare that the research was conducted in the absence of any commercial or financial relationships that could be construed as a potential conflict of interest.

## Supplementary Material

Supplementary Figure 1 Weighted gene co-expression network analysis of Rongchang pigs. **(A)** Analysis of network topology of Rongchang pig showed that it met the scale-free topology threshold of 0.8 when β = 20. The left panel shows the scale-free fit index as a function of the soft-threshold power. The right panel displays the mean connectivity as a function of the soft-threshold power. **(B)** The dissimilarity was based on topological overlap. The “Merged dynamic” is the result of merging modules with a correlation higher than 0.9. The y-axis is the distance determined by the extent of topological overlap. **(C)** Heatmap displaying the correlations and significant differences between gene modules and six tissues of Rongchang pigs. Red represents high adjacency (positive correlation) and blue represents low adjacency (negative correlation). In brackets is the *p*-value of the correlation test.

Supplementary Figure 2 The co-expression network of six tissues key modules of Tibetan pig. The co-expression network of muscle, liver, heart, spleen, kidney and lung in the figure shows the co-expression relationship of weight above 0.35, 0.35, 0.25, 0.35, 0.35, and 0.25, respectively. The dark blue circles in the figure represent the hub genes of each network.

Supplementary Table 1 List of genes in the key modules of the six tissues of Tibetan pigs and Rongchang pigs.

Supplementary Table 2 List of tissue-specific genes for Tibetan pigs and Rongchang pigs. The first two columns in the table are the gene name and Ensembl ID. Columns 3–5 are the position coordinates of the gene. Columns 6–7 are information about the tissue-specific genes of Tibetan pigs, and the seventh column represents the TSI value of the Tibetan pig TS genes. Columns 8–9 are the information of Rongchang pig TS genes, and the ninth column represents the TSI value of the Rongchang pig TS genes.

Supplementary Table 3 Significantly enriched signaling pathways in key modules of various tissues in Tibetan pigs and Rongchang pigs.

Supplementary Table 4 Pathways that are only significantly enriched in Rongchang pig tissue modules.

Supplementary Table 5 Correlation coefficients and *p*-values between candidate genes in the three validation groups (Diqing Tibetan pig, Tibetan sheep and yak).
