# Supplementary figures and images for: KLF4, a Key Regulator of a Transitive Triplet, Acts on the TGF-β Signaling Pathway and Contributes to High-Altitude Adaptation of Tibetan Pigs
Source: Front Genet. 2021 Apr 15;12:628192. doi: 10.3389/fgene.2021.628192 (PMC8082500; doi:10.3389/fgene.2021.628192)

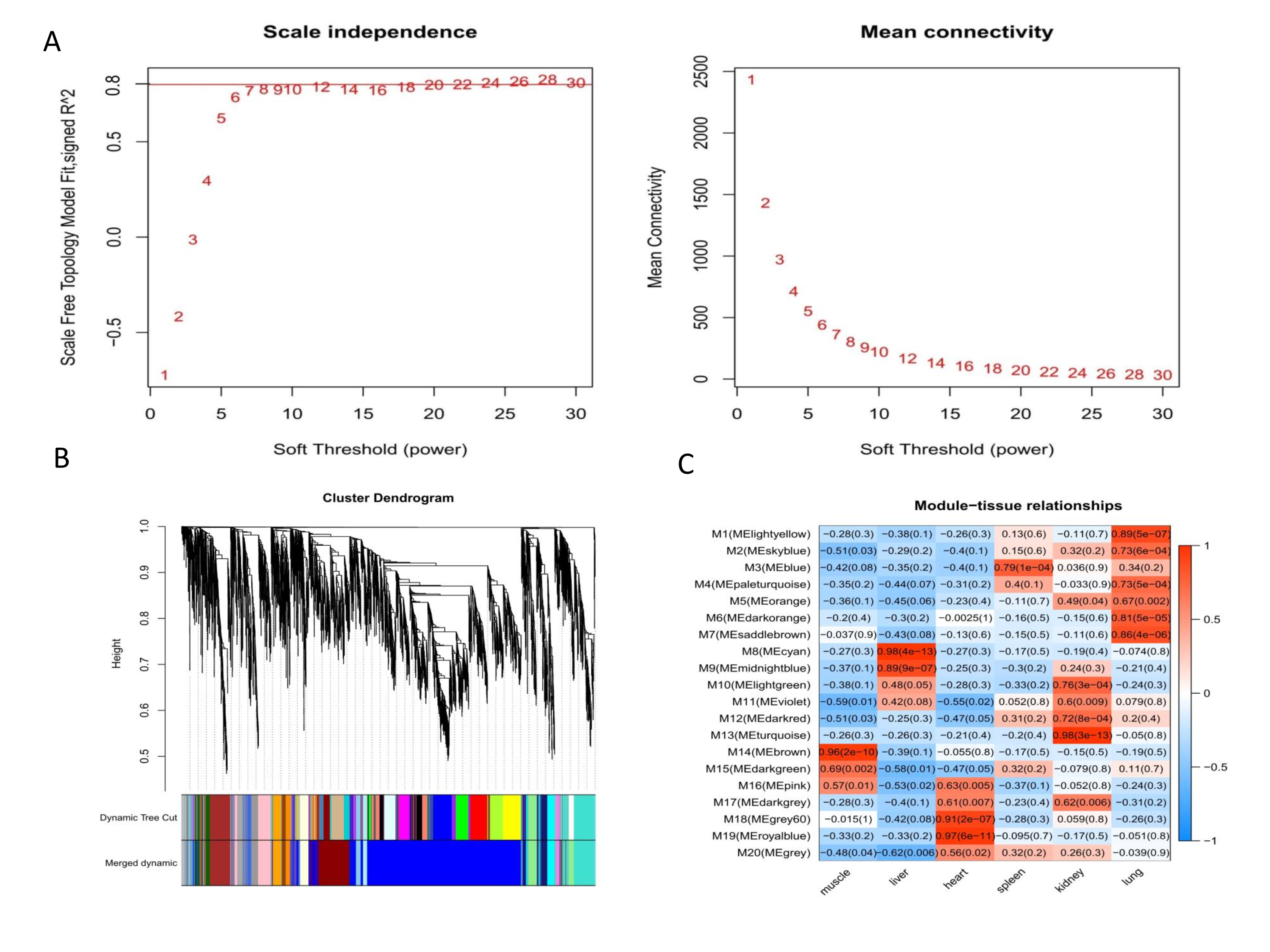

Supplement: Supplementary Figure 1 — Weighted gene co-expression network analysis of Rongchang pigs. (A) Analysis of network topology of Rongchang pig showed that it met the scale-free topology threshold of 0.8 when β = 20. The left panel shows the scale-free fit index as a function of the soft-threshold power. The right panel displays the mean connectivity as a function of the soft-threshold power. (B) The dissimilarity was based on topological overlap. The “Merged dynamic” is the result of merging modules with a correlation higher than 0.9. The y-axis is the distance determined by the extent of topological overlap. (C) Heatmap displaying the correlations and significant differences between gene modules and six tissues of Rongchang pigs. Red represents high adjacency (positive correlation) and blue represents low adjacency (negative correlation). In brackets is the p-value of the correlation test. [file Image_1.TIF]

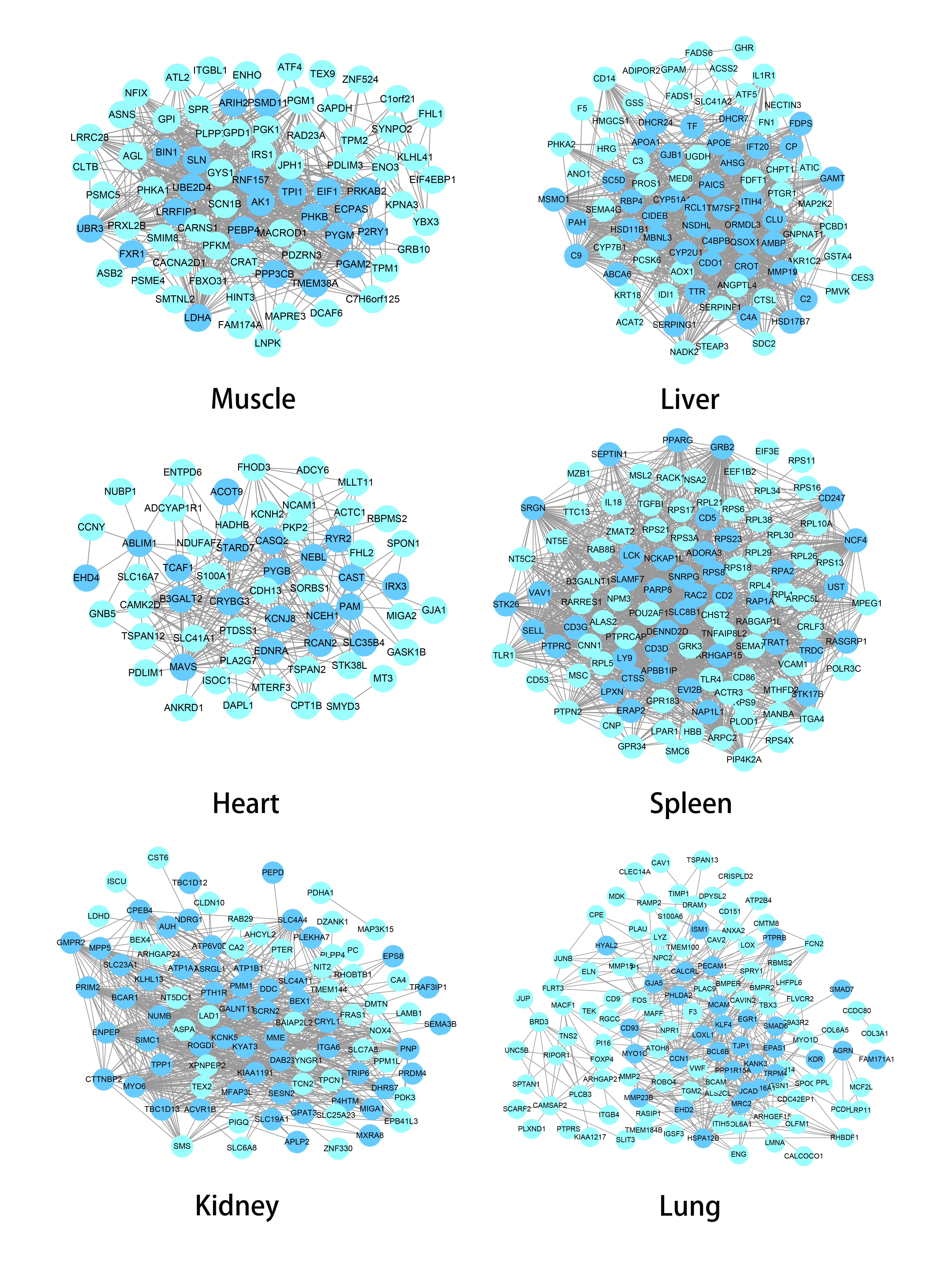

Supplement: Supplementary Figure 2 — The co-expression network of six tissues key modules of Tibetan pig. The co-expression network of muscle, liver, heart, spleen, kidney and lung in the figure shows the co-expression relationship of weight above 0.35, 0.35, 0.25, 0.35, 0.35, and 0.25, respectively. The dark blue circles in the figure represent the hub genes of each network. [file Image_2.JPEG]
