# Supplementary material for: KLF4, a Key Regulator of a Transitive Triplet, Acts on the TGF-β Signaling Pathway and Contributes to High-Altitude Adaptation of Tibetan Pigs
Source: Front Genet. 2021 Apr 15;12:628192. doi: 10.3389/fgene.2021.628192 (PMC8082500; doi:10.3389/fgene.2021.628192)
Supplement: Supplementary Table 3 — Significantly enriched signaling pathways in key modules of various tissues in Tibetan pigs and Rongchang pigs. [file Table_3.DOCX]

**Supplementary table S3. Significantly enriched signaling pathways in key modules of various tissues in Tibetan pigs and Rongchang pigs.**

| **Pig_breeds** | **Tissues** | **Category** | **GO/KEGG ID** | **Term** | **P-value** | **Benjamini** |
| --- | --- | --- | --- | --- | --- | --- |
| Tibetan pig | Muscle | Biological Progresses | GO:0006096 | Glycolytic process | 9.92E-06 | 6.21E-03 |
|  |  | Cellular Components | GO:0031595 | Nuclear proteasome complex | 1.02E-04 | 3.97E-03 |
|  |  |  | GO:0008540 | Proteasome regulatory particle, base subcomplex | 4.61E-04 | 1.02E-2 |
|  |  | KEGG_PATHWAY | ssc04922 | Glucagon signaling pathway | 3.76E-08 | 3.26E-06 |
|  |  |  | ssc00010 | Glycolysis / gluconeogenesis | 2.66E-06 | 1.53E-04 |
|  |  |  | ssc03050 | Proteasome | 7.28E-06 | 3.15E-04 |
|  |  |  | ssc01130 | Biosynthesis of antibiotics | 6.35E-05 | 1.83E-03 |
|  |  |  | ssc01230 | Biosynthesis of amino acids | 5.63E-05 | 1.95E-03 |
|  |  |  | ssc01200 | Carbon metabolism | 2.70E-04 | 6.64E-03 |
|  |  |  | ssc04152 | AMPK signaling pathway | 6.39E-04 | 1.37E-03 |
|  |  |  | ssc04720 | Long-term potentiation | 7.61E-04 | 1.45E-02 |
|  |  |  | ssc04261 | Adrenergic signaling in cardiomyocytes | 1.27E-03 | 2.18E-02 |
|  |  |  | ssc04931 | Insulin resistance | 2.35E-03 | 3.63E-02 |
|  |  |  | ssc05169 | Epstein-Barr virus infection | 2.60E-03 | 3.69E-02 |
|  |  |  | ssc04722 | Neurotrophin signaling pathway | 3.16E-03 | 3.83E-02 |
|  |  |  | ssc04921 | Oxytocin signaling pathway | 3.15E-03 | 4.11E-02 |
|  | Liver | Biological Progresses | GO:0006956 | Complement activation | 1.23E-04 | 4.34E-02 |
|  |  | KEGG_PATHWAY | ssc04610 | Complement and coagulation cascades | 1.32E-07 | 5.78E-06 |
|  | Heart | KEGG_PATHWAY | ssc05412 | Arrhythmogenic right ventricular cardiomyopathy  (ARVC) | 1.36E-04 | 1.85E-02 |
|  | Spleen | Biological Progresses | GO:0006412 | Translation | 1.18E-17 | 6.86E-15 |
|  |  |  | GO:0001731 | Formation of translation preinitiation complex | 4.56E-05 | 1.31E-02 |
|  |  |  | GO:0006446 | Regulation of translational initiation | 9.43E-05 | 1.81E-02 |
|  |  | Cellular Components | GO:0022627 | Cytosolic small ribosomal subunit | 3.57E-19 | 6.49E-17 |
|  |  |  | GO:0022625 | Cytosolic large ribosomal subunit | 1.48E-09 | 8.99E-08 |
|  |  |  | GO:0016282 | Eukaryotic 43S preinitiation complex | 4.96E-06 | 1.29E-04 |
|  |  |  | GO:0033290 | Eukaryotic 48S preinitiation complex | 7.72E-06 | 1.76E-04 |
|  |  |  | GO:0005852 | Eukaryotic translation initiation factor 3 complex | 1.08E-04 | 2.17E-03 |
|  |  |  | GO:0034709 | Methylosome | 2.26E-04 | 4.11E-03 |
|  |  |  | GO:0005683 | U7 snrnp | 1.56E-03 | 2.34E-02 |
|  |  |  | GO:0042105 | Alpha-beta T cell receptor complex | 1.56E-03 | 2.35E-02 |
|  |  | Molecular Function | GO:0003735 | Structural constituent of ribosome | 9.11E-21 | 1.59E-18 |
|  |  |  | GO:0003743 | Translation initiation factor activity | 1.65E-04 | 9.59E-03 |
|  |  | KEGG_PATHWAY | ssc03010 | Ribosome | 2.54E-21 | 4.10E-19 |
|  | Lung | KEGG_PATHWAY | ssc04510 | Focal adhesion | 2.45E-07 | 3.69E-05 |
|  |  |  | ssc04512 | ECM-receptor interaction | 3.95E-06 | 2.98E-04 |
|  |  |  | ssc04151 | PI3K-Akt signaling pathway | 4.93E-04 | 0.009256 |
|  |  |  | ssc04350 | TGF-beta signaling pathway | 1.20E-03 | 1.80E-02 |
| Rongchang pig | Muscle | Biological Progresses | GO0006096 | Glycolytic process | 1.73E-06 | 1.32E-03 |
|  |  | KEGG_PATHWAY | ssc04922 | Glucagon signaling pathway | 3.09E-08 | 2.81E-06 |
|  |  |  | ssc00010 | Glycolysis / gluconeogenesis | 9.58E-07 | 5.81E-05 |
|  |  |  | ssc01130 | Biosynthesis of antibiotics | 1.24E-03 | 3.17E-02 |
|  |  |  | ssc01230 | Biosynthesis of amino acids | 1.16E-3 | 3.44E-02 |
|  |  |  | ssc04720 | Long-term potentiation | 1.89E-03 | 4.20E-02 |
|  | Liver | Biological Progresses | GO:0006956 | Complement and coagulation cascades | 1.66E-06 | 1.18E-03 |
|  |  | KEGG_PATHWAY | ssc04610 | Complement activation | 7.94E-09 | 6.15E-07 |
|  | Heart | Cellular Components | GO:0005747 | Mitochondrial respiratory chain complex I | 3.71E-14 | 5.90E-12 |
|  |  |  | GO:0005753 | Mitochondrial proton-transporting ATP synthase complex | 3.67E-05 | 1.17E-03 |
|  |  | KEGG_PATHWAY | ssc05012 | Parkinson's disease | 4.54E-26 | 3.76E-24 |
|  |  |  | ssc00190 | Oxidative phosphorylation | 3.03E-26 | 5.03E-24 |
|  |  |  | ssc05016 | Huntington's disease | 1.77E-23 | 9.77E-22 |
|  |  |  | ssc05010 | Alzheimer's disease | 9.78E-23 | 4.06E-21 |
|  |  |  | ssc04932 | Non-alcoholic fatty liver disease (NAFLD) | 5.75E-19 | 1.91E-17 |
|  |  |  | ssc01100 | Metabolic pathways | 1.62E-12 | 4.49E-11 |
|  |  |  | ssc00020 | Citrate cycle (TCA cycle) | 8.34E-05 | 1.73E-03 |
|  |  |  | ssc01200 | Carbon metabolism | 1.01E-03 | 1.83E-02 |
|  |  |  | ssc01130 | Biosynthesis of antibiotics | 2.65E-03 | 4.30E-02 |
|  | Spleen | Biological Progresses | GO:0034314 | Arp2/3 complex-mediated actin nucleation | 1.18E-06 | 1.26E-03 |
|  |  | Cellular Components | GO:0005885 | Arp2/3 protein complex | 4.17E-08 | 4.70E-06 |
|  |  |  | GO:0005687 | U4 snrnp | 8.45E-04 | 3.13E-02 |
|  |  |  | GO:0034709 | Methylosome | 1.35E-03 | 4.57E-02 |
|  |  |  | GO:0071013 | Catalytic step 2 spliceosome | 1.62E-03 | 4.77E-02 |
|  |  | KEGG_PATHWAY | ssc05140 | Leishmaniasis | 5.41E-09 | 6.49E-07 |
|  |  |  | ssc05145 | Toxoplasmosis | 3.61E-07 | 1.73E-05 |
|  |  |  | ssc05152 | Tuberculosis | 5.42E-06 | 1.45E-04 |
|  |  |  | ssc05323 | Rheumatoid arthritis | 2.37E-05 | 3.55E-04 |
|  |  |  | ssc04612 | Antigen processing and presentation | 7.44E-05 | 9.39E-04 |
|  |  |  | ssc04672 | Intestinal immune network for iga production | 1.18E-04 | 1.35E-03 |
|  |  |  | ssc05321 | Inflammatory bowel disease (IBD) | 5.90E-04 | 5.65E-03 |
|  |  |  | ssc05416 | Viral myocarditis | 6.81E-04 | 6.27E-03 |
|  |  |  | ssc05150 | Staphylococcus aureus infection | 1.00E-03 | 8.58E-03 |
|  |  |  | ssc05310 | Asthma | 1.49E-03 | 1.15E-02 |
|  |  |  | ssc05220 | Chronic myeloid leukemia | 1.70E-03 | 1.23E-02 |
|  |  |  | ssc05132 | Salmonella infection | 2.04E-03 | 1.43E-02 |
|  |  |  | ssc05161 | Hepatitis B | 2.36E-03 | 1.56E-02 |
|  |  |  | ssc05212 | Pancreatic cancer | 9.14E-03 | 4.78E-02 |
