# Supplementary material for: KLF4, a Key Regulator of a Transitive Triplet, Acts on the TGF-β Signaling Pathway and Contributes to High-Altitude Adaptation of Tibetan Pigs
Source: Front Genet. 2021 Apr 15;12:628192. doi: 10.3389/fgene.2021.628192 (PMC8082500; doi:10.3389/fgene.2021.628192)
Supplement: Supplementary Table 4 — Pathways that are only significantly enriched in Rongchang pig tissue modules. [file Table_4.DOCX]

**Supplementary table S4. Pathways that are only significantly enriched in Rongcahng pig tissue modules.**

| **Tissue** | **Category** | **GO/KEGG** | **Trem** | **P-value** | **Benjamini** |
| --- | --- | --- | --- | --- | --- |
| Heart | Cellular Components | GO:0005747 | Mitochondrial respiratory chain complex I | 3.71E-14 | 5.90E-12 |
|  |  | GO:0005753 | Mitochondrial proton-transporting ATP synthase complex | 3.67E-05 | 1.17E-03 |
|  | KEGG_PATHWAY | ssc05012 | Parkinson's disease | 4.54E-26 | 3.76E-24 |
|  |  | ssc00190 | Oxidative phosphorylation | 3.03E-26 | 5.03E-24 |
|  |  | ssc05016 | Huntington's disease | 1.77E-23 | 9.77E-22 |
|  |  | ssc05010 | Alzheimer's disease | 9.78E-23 | 4.06E-21 |
|  |  | ssc04932 | Non-alcoholic fatty liver disease (NAFLD) | 5.75E-19 | 1.91E-17 |
|  |  | ssc01100 | Metabolic pathways | 1.62E-12 | 4.49E-11 |
|  |  | ssc00020 | Citrate cycle (TCA cycle) | 8.34E-05 | 1.73E-03 |
|  |  | ssc01200 | Carbon metabolism | 1.01E-03 | 1.84E-03 |
|  |  | ssc01130 | Biosynthesis of antibiotics | 2.65e-03 | 4.30E-03 |
| Spleen | Biological Progresses | GO:0034314 | Arp2/3 complex-mediated actin nucleation | 1.18E-06 | 1.26E-03 |
|  | Cellular Components | GO:0005885 | Arp2/3 protein complex | 4.17E-08 | 4.70E-06 |
|  |  | GO:0005687 | U4 snrnp | 8.45E-04 | 3.13E-03 |
|  |  | GO:0034709 | Methylosome | 1.35E-03 | 4.57E-03 |
|  |  | GO:0071013 | Catalytic step 2 spliceosome | 1.62E-03 | 4.77E-03 |
|  | KEGG_PATHWAY | ssc05140 | Leishmaniasis | 5.41E-09 | 6.49E-07 |
|  |  | ssc05145 | Toxoplasmosis | 3.61E-07 | 1.73E-05 |
|  |  | ssc05152 | Tuberculosis | 5.42E-06 | 1.45E-04 |
|  |  | ssc05323 | Rheumatoid arthritis | 2.37E-05 | 3.55E-04 |
|  |  | ssc04612 | Antigen processing and presentation | 7.44E-05 | 9.39E-04 |
|  |  | ssc04672 | Intestinal immune network for iga production | 1.18E-04 | 1.35E-03 |
|  |  | ssc05321 | Inflammatory bowel disease (IBD) | 5.90E-04 | 5.65E-03 |
|  |  | ssc05416 | Viral myocarditis | 6.81E-04 | 6.23E-03 |
|  |  | ssc05150 | Staphylococcus aureus infection | 1.00E-03 | 8.58E-03 |
|  |  | ssc05310 | Asthma | 1.49E-03 | 1.15E-03 |
|  |  | ssc05220 | Chronic myeloid leukemia | 1.70E-03 | 1.23E-03 |
|  |  | ssc05132 | Salmonella infection | 2.04E-03 | 1.43E-03 |
|  |  | ssc05161 | Hepatitis B | 2.36E-03 | 1.56E-02 |
|  |  | ssc05212 | Pancreatic cancer | 9.14E-03 | 4.78E-02 |
