# Supplementary material for: KLF4, a Key Regulator of a Transitive Triplet, Acts on the TGF-β Signaling Pathway and Contributes to High-Altitude Adaptation of Tibetan Pigs
Source: Front Genet. 2021 Apr 15;12:628192. doi: 10.3389/fgene.2021.628192 (PMC8082500; doi:10.3389/fgene.2021.628192)
Supplement: Supplementary Table 5 — Correlation coefficients and p-values between candidate genes in the three validation groups (Diqing Tibetan pig, Tibetan sheep and yak). [file Table_5.DOCX]

**Supplementary table S5. Correlation coefficients and p-values between candidate genes in the three validation groups (Diqing Tibetan pig, Tibetan sheep and yak).**

| **Validation groups** | **Gene A** | **Gene B** | **Correlation coefficient** | **p-value** |
| --- | --- | --- | --- | --- |
| Diqing Tibetan pig | KLF4 | EGR1 | 0.9961 | **3.92E-03** |
|  | KLF4 | EPAS1 | 0.9965 | **3.51E-03** |
|  | KLF4 | SMAD6 | 0.9976 | **2.43E-03** |
|  | KLF4 | SMAD7 | 0.9955 | **4.54E-03** |
|  | KLF4 | KDR | 0.0373 | 9.63E-01 |
|  | EGR1 | EPAS1 | 0.9995 | **5.00E-04** |
|  | EGR1 | SMAD6 | 0.9991 | **9.01E-04** |
|  | EGR1 | SMAD7 | 0.9843 | **1.57E-02** |
|  | EGR1 | KDR | 0.0279 | 9.72E-01 |
|  | EPAS1 | SMAD6 | 0.9998 | **2.03E-04** |
|  | EPAS1 | SMAD7 | 0.9870 | **1.30E-02** |
| Tibetan sheep | KLF4 | BCL6B | 0.9399 | **7.12E-09** |
|  | KLF4 | EPAS1 | 0.7324 | **5.01E-04** |
|  | KLF4 | SMAD6 | 0.7432 | **4.03E-04** |
|  | KLF4 | KDR | 0.6973 | **1.32E-03** |
|  | KLF4 | CCN1 | 0.7400 | **4.01E-04** |
|  | KLF4 | EGR1 | 0.5626 | **1.51E-02** |
|  | KLF4 | ATOH8 | 0.8074 | **5.10E-05** |
|  | EGR1 | BCL6B | 0.4185 | 8.39E-02 |
|  | EGR1 | EPAS1 | 0.2989 | 2.28E-01 |
|  | EGR1 | SMAD6 | 0.2821 | 2.57E-01 |
|  | EGR1 | KDR | 0.3228 | 1.91E-01 |
|  | EGR1 | CCN1 | 0.8297 | **2.06E-05** |
|  | EGR1 | ATOH8 | 0.09710 | 4.03E-01 |
|  | BCL6B | EPAS1 | 0.6193 | **6.13E-03** |
|  | BCL6B | SMAD6 | 0.6083 | **7.42E-03** |
|  | BCL6B | KDR | 0.5783 | **1.19E-02** |
|  | BCL6B | CCN1 | 0.5830 | **1.11E-02** |
|  | BCL6B | ATOH8 | 0.8514 | **7.41E-06** |
|  | EPAS1 | SMAD6 | 0.9603 | **2.72E-10** |
| Yak | KLF4 | BCL6B | 0.8477 | **8.93E-06** |
|  | KLF4 | EPAS1 | 0.7086 | **1.01E-03** |
|  | KLF4 | SMAD6 | 0.6138 | **6.73E-03** |
|  | KLF4 | SMAD7 | 0.5188 | **2.74E-02** |
|  | KLF4 | CCN1 | 0.7705 | **2.03E-04** |
|  | KLF4 | EGR1 | 0.3604 | 1.42E-01 |
|  | KLF4 | KDR | 0.2098 | 4.03E-01 |
|  | EGR1 | BCL6B | 0.0585 | 8.18E-01 |
|  | EGR1 | EPAS1 | -0.1829 | 4.67E-01 |
|  | EGR1 | SMAD6 | -0.1616 | 5.22E-01 |
|  | EGR1 | SMAD7 | -0.1622 | 5.20E-01 |
|  | EGR1 | CCN1 | 0.7965 | **3.27E-02** |
|  | EGR1 | KDR | 0.4327 | 1.97E-01 |
|  | BCL6B | EPAS1 | 0.7192 | **8.04E-04** |
|  | BCL6B | SMAD6 | 0.6092 | **7.32E-03** |
|  | BCL6B | SMAD7 | 0.5603 | **1.56E-02** |
|  | BCL6B | CCN1 | 0.6178 | **6.32E-03** |
|  | BCL6B | KDR | 0.3714 | 1.29E-01 |
|  | EPAS1 | SMAD6 | 0.9350 | **1.30E-08** |
|  | EPAS1 | SMAD7 | 0.9163 | **9.33E-08** |
