# Supplementary material for: KLF4, a Key Regulator of a Transitive Triplet, Acts on the TGF-β Signaling Pathway and Contributes to High-Altitude Adaptation of Tibetan Pigs
Source: Front Genet. 2021 Apr 15;12:628192. doi: 10.3389/fgene.2021.628192 (PMC8082500; doi:10.3389/fgene.2021.628192)
Supplement: Supplementary file 8 [file Data_Sheet_1.docx]

***KLF4*, a Key Regulator of a Transitive Triplet, Acts on the TGF-β Signaling Pathway and Contributes to High-Altitude Adaptation of Tibetan Pigs**

**Supplementary materials**

**1. Technical roadmap of this research**


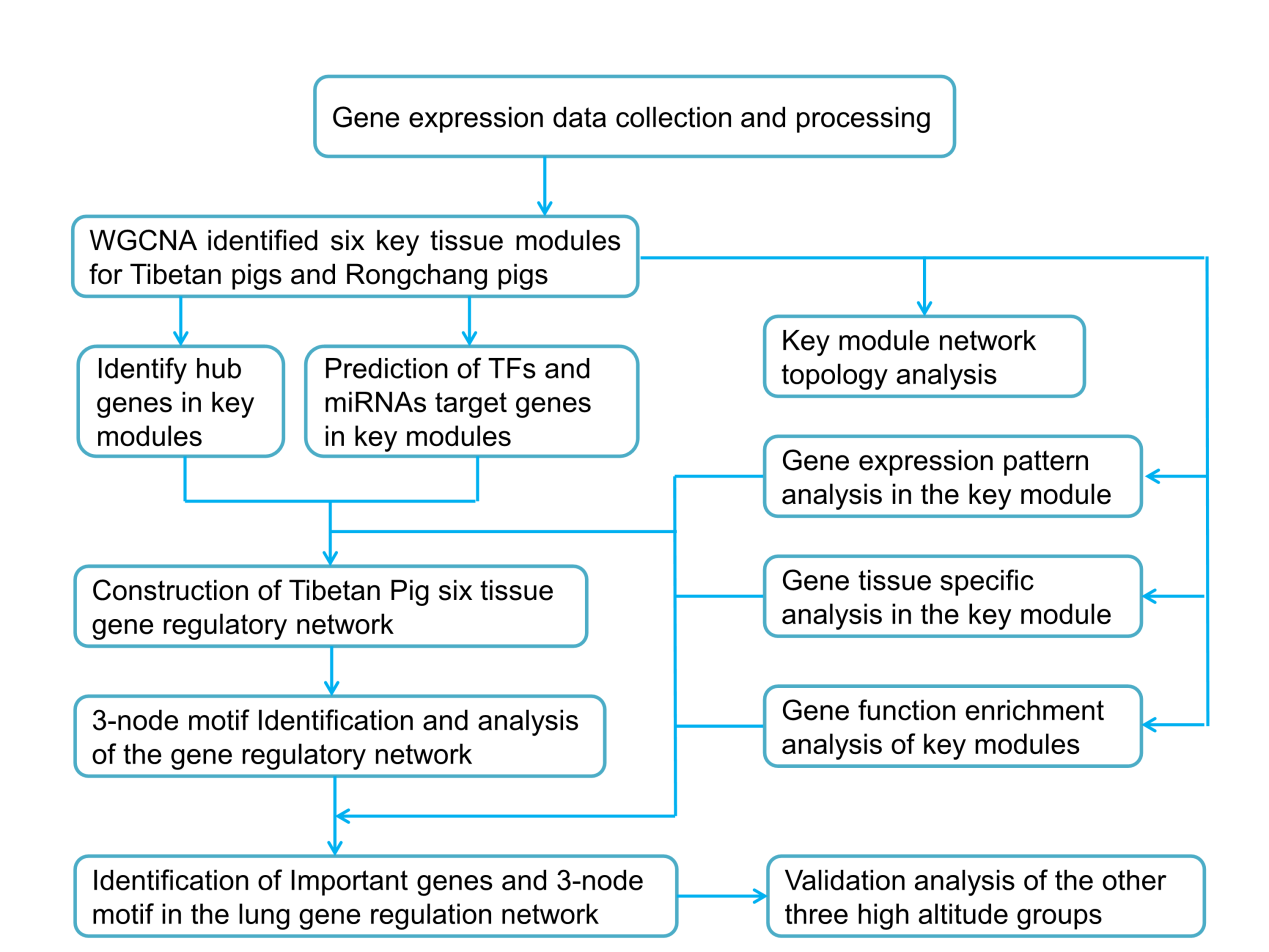


**2. Experimental animals**

According to the introduction of Tang et al. (2017) and long et al. (2019), Tibetan pigs were collected from Songpan County, Tibet, China, where the altitude is more than 3000 meters, the oxygen content in the air is far lower than the normal state, and the oxygen partial pressure in the air is about 14.69%. Rongchang pigs were collected from Chongqing, China, where the altitude is about 400m, and the oxygen content in the atmosphere is similar to that of normal oxygen. It is cross-fertile relatives in a low-altitude region that is geographically adjacent to Tibetan pigs.

**3. The details of the data analysis**

**3.1 Co-expression Network Analysis**

Based on gene expression data, we used WGCNA to construct a co-expression network for Tibetan pigs and Rongchang pigs. First, cluster analysis of Tibetan pig and Rongchang pig samples based on the hierarchical clustering algorithm, using the “hclust” function in WGCNA was used to verify the clustering of samples and to detect outliers. Then, the soft threshold power β was obtained to meet the scale-free topology criterion (Zhang et al. 2005). Based on β, the Pearson correlation matrix between genes was transformed into an adjacency matrix:

$\alpha_{\mathrm{ij}}=\left| r_{\mathrm{ij}} \right|^{\beta}$ [1]

The topological overlap measure (TOM) representing the overlap in shared neighbors was calculated using the adjacency matrix:

$\mathrm{TOM}_{i,j}=\frac{\sum_{K=1}^{N} \alpha_{i,k}\cdot\alpha_{k,j}+\alpha_{i,j}}{\min\left( K_{i},K_{j} \right)+1-\alpha_{i,j}}$ [2]

where $\alpha$ is the adjacency matrix given by formula [1]. Based on the TOM matrix, genes with similar expression profiles were classified into the same modules using hierarchical clustering and dynamic branch cutting procedures. Relationships between modules can be studied using the correlation between module eigengenes. Here, we merged modules with a correlation higher than 0.9. The number of genes in the merged module should be more than 50.

Gene significance (GS)is a parameter to characterize the correlation between genes and phenotypic traits. The higher the absolute value of ${GS}_{i}$is, the more biologically significant the i-th gene (Langfelder et al. 2008).We used the following criteria to identify the key module of each tissue: (1) the p-value of the correlation between the module and the tissue was less than threshold $3.97\times{10}^{-4}$ (0.05/126) using Bonferroni correction method; and (2) the median of the GS value was greater than 0.8.

Finally, we calculated the fundamental topology concepts of each key module, including density, mean cluster coefficient, centralization and heterogeneity.

**3.2 Gene Tissue-specific Analysis**

We used the tissue-specificity index (TSI, τ) (Yanai et al. 2005) to grade the scalar measure of the specificity of an expression profile, which ranged from 0 for housekeeping genes to 1 for strictly TS genes. The index τ was defined as follows:

$\tau=\frac{\sum_{i=1}^{N} \left( 1-x_{i} \right)}{N-1}$ [3]

where *N* is the number of tissues and $x_{i}$is the expression normalized by the maximal component value. According to Yania et al. (2005), genes with TSI> 0.9 were considered TS genes.

**3.3 Identification of Hub Genes in Key Modules**

For each module, Langfelder et al. (2008) define a quantitative measure of module membership (MM) as the correlation of the module eigengenes and the gene expression profile. The MM of gene i in module q can be defined as follows:

$\mathrm{MM}^{q}=K_{cor, i}^{(q)}=cor\left( x_{i},E^{(q)} \right)$ [4]

where $x_{i}$is the profile of gene i and $E^{(q)}$ is the module eigengene of module q. If ${MM}_{i}^{q}$ is close to 0, the i-th gene is not part of the q module. On the other hand, if ${MM}_{i}^{q}$ is close to 1 or -1, it is highly connected to the q module genes.

In co-expression networks, the connectivity ($k_{i}$) is defined as the sum of connection strengths with the other genes:

$k_{i}=\sum_{\mu\neq i} a_{\mu i}$ [5]

The Kwithin of a gene is the sum of the connectivity of this gene in the module.

We identified the hub genes in each key module according to the following criteria: (1) GS value of the gene ≥ 0.8; (2) MM value of the gene ≥ 0.95; and (3) in each module, Kwithin ranked in the top 20% of genes.

**3.4 Gene Regulatory Network Construction**

First, we removed the co-expression relationship with a weight value of less than 0.2 in the network of the six key modules of Tibetan pigs. Using the AnimalTFDB database (Hu et al. 2019), we obtained the TFs in each key module. The biomaRt package of R (Durinck et al. 2009) was used to obtain the sequence of the 1 kb region upstream of the transcription start site of all protein-encoding genes in the pig genome. The TF position weight matrix (PWM) of pigs was obtained from the CIS-BP database (Weirauch et al. 2014). Using the TFBSTools package in R (Tan et al. 2016) to predict the target genes of TFs, the relScore value was set to 0.85, and other parameters were defaulted. Next, the biomaRt package was used to obtain the sequence of the 3’UTR region of pig protein coding protein genes. We obtained all mature miRNA sequences from the miRBase database (Kozomara et al. 2019). Based on the miRanda tool (Enright et al. 2003), we predicted target genes of the miRNAs, and the Tot Score and Tot Energy were set to 140 and -20, respectively. Finally, the gene regulatory network in each Tibetan pig tissue was constructed by combining TFs, miRNAs, target genes, co-expressed genes, hub genes and their interactions.

**3.5 Motif Analysis of the Gene Regulatory Network**

Gene networks may contain various subgraphs, and the detection of motifs contributes to identifying the typical local connection pattern (Milo et al. 2002, Ravasz et al. 2002, Bascompte. 2009, Alon. 2007). The 3-node motifs in the gene regulatory network of each tissue were obtained using mfinder1.2 (Kashtan et al. 2004). Mfinder1.2 implements a switching method to generate random network, which can switch between edges while maintaining the number of incoming edges, outgoing edges and mutual edges of each node of the input network. In this study, the number of random networks was set to 10000. Moreover, mfinder1.2 describes the significance of the difference between the frequency of motifs in the real network and that in the corresponding randomized network using the Z-test in statistics. The Z-test is defined as follows:

$Z_{j}\triangleq Z\left( j \right)=\frac{N\left（ j \right）-\overline{N_{r} (j)}}{\sigma_{r}(j)}$ [6]

where $N\left（ j \right）$ is the number of times the subgraph appears in the real network, and $\overline{N_{r}(j)}$ and $\sigma_{r}(j)$ are the mean and standard deviation of its appearances in the randomized network ensemble. The larger the absolute value of Z is, the more significant the difference. The significance profile (SP) is the vector of Z scores normalized to length 1, describing the statistical significance of each motif in the network (Milo et al. 2004):

$\mathrm{SP}_{i}=\frac{Z_{i}}{\left( \sum Z_{i}^{2} \right)^{1/2}}$ [7]

We constructed the triad significance profile (TSP) of the six tissues from Tibetan pigs, which display certain relations between subgraph types.

**3.6 Identification of Important Genes and** **Size-3 Subgraphs in the Lung-specific Gene Regulation Network**

Each node was scored according to the connectivity, differential expression between different conditions, tissue-specific expression and TF characteristics using the following formula [8]:

$S_{\mathrm{node}_{i}}=\omega_{i}K_{i}q_{i}\mathrm{TSI}_{i}$ $\omega=\left\{ \begin{aligned} &0.5 the number of TG of TF\geq\overline{TGs} \\ &0.3 the number of TG of TF<\overline{TGs} \\ &0.2 N\mathrm{on}TFs \end{aligned} \right.$ [8]

Where $K_{i}$is the scaled connectivity of each gene in the regulatory network, and $K_{i}$ of the i-th node is defined as follows:

$K_{i}=\frac{\mathrm{Connectivity}_{i}}{\max\left( \mathrm{Connectivity} \right)}$ [9]

$q_{i}$ is the estimated probability of differentially expressed genes in lung tissues between Tibetan pigs and Rongchang pigs, calculated by NOISeq (Tarazona et al. 2015); ${TSI}_{i}$ is the tissue-specificity index of the gene; and $\omega_{i}$ is the weighting coefficient. $\overline{TGs}$ is the average number of target genes regulated by TFs. If the TF regulated more than$\overline{TGs}$, ω is set to 0.5, the target gene is less than $\overline{TGs}$, ω is set to 0.3. The ω of non-TF genes is set to 0.2.

The score of each candidate size-3 subgraph was calculated by combining the node score and the edge score as follows:

$S_{\mathrm{motif}_{i}}=\frac{\sum_{node\in motif} S_{\mathrm{node}}}{\sqrt{n_{\mathrm{node}}}}+\frac{\sum_{edge\in motif} S_{\mathrm{edge}}}{\sqrt{n_{\mathrm{edge}}}}$ [10]

where $S_{\mathrm{edge}}$ denotes the score of each edge, which was the weight value of the edge from WGCNA, and $n_{\mathrm{node}}$ and $n_{\mathrm{edge}}$ are the number of nodes and edges in the motif, respectively.

**3.7 Verification of Important Genes in Lung Tissue**

The lung tissue expression profiles of 3 Tibetan sheep and 3 yaks was obtained from the GEO database (accession: GSE93855) (Tang et al. 2017), the expression profiles of 4 Diqing Tibetan pigs lung tissue from another dataset (accession: GSE84409) (Jia et al. 2016), and WGCNA was performed. The Hmisc package in R was used to statistically test the correlation between genes.

# REFERENCES

Tang Q, Gu Y, Zhou X, Jin L, Guan J, Liu R, et al. Comparative transcriptomics of 5 high-altitude vertebrates and their low-altitude relatives. Gigascience. 2017; 6(12):1-9. doi: 10.1093/gigascience/gix105.

Long K, Feng S, Ma J, Zhang J, Jin L, Tang Q, et al. Small non-coding RNA transcriptome of four high-altitude vertebrates and their low-altitude relatives. Sci Data. 2019 Oct 4;6(1):192. doi: 10.1038/s41597-019-0204-5.

Langfelder P, Horvath S. WGCNA: an R package for weighted correlation network analysis. BMC Bioinformatics. 2008; 9:559. doi: 10.1186/1471-2105-9-559.

Zhang B, Horvath S. A general framework for weighted gene co-expression network analysis. Stat Appl Genet Mol Biol. 2005; 4:Article17. doi: 10.2202/1544-6115.1128.

Yanai I, Benjamin H, Shmoish M, Chalifa-Caspi V, Shklar M, Ophir R, et al. Genome-wide midrange transcription profiles reveal expression level relationships in human tissue specification. Bioinformatics. 2005; 21(5):650-9. doi: 10.1093/bioinformatics/bti042.

Hu H, Miao YR, Jia LH, Yu QY, Zhang Q, Guo AY. AnimalTFDB 3.0: a comprehensive resource for annotation and prediction of animal transcription factors. Nucleic Acids Res. 2019; 47(D1):D33-D38. doi: 10.1093/nar/gky822.

Durinck S, Spellman PT, Birney E, Huber W. Mapping identifiers for the integration of genomic datasets with the R/Bioconductor package biomaRt. Nat Protoc. 2009; 4(8):1184-91. doi: 10.1038/nprot.2009.97.

Weirauch MT, Yang A, Albu M, Cote AG, Montenegro-Montero A, Drewe P, et al. Determination and inference of eukaryotic transcription factor sequence specificity. Cell. 2014; 158(6):1431-1443. doi: 10.1016/j.cell.2014.08.009.

Tan G, Lenhard B. TFBSTools: an R/bioconductor package for transcription factor binding site analysis. Bioinformatics. 2016; 32(10):1555-6. doi: 10.1093/bioinformatics/btw024.

Kozomara A, Birgaoanu M, Griffiths-Jones S. miRBase: from microRNA sequences to function. Nucleic Acids Res. 2019; 47(D1):D155-D162. doi: 10.1093/nar/gky1141.

Enright AJ, John B, Gaul U, Tuschl T, Sander C, Marks DS. MicroRNA targets in Drosophila. Genome Biol. 2003; 5(1):R1. doi: 10.1186/gb-2003-5-1-r1.

Milo R, Itzkovitz S, Kashtan N, Levitt R, Shen-Orr S, Ayzenshtat I, et al. Superfamilies of evolved and designed networks. Science. 2004; 303(5663):1538-42. doi: 10.1126/science.1089167.

Milo R, Shen-Orr S, Itzkovitz S, Kashtan N, Chklovskii D, Alon U. Network motifs: simple building blocks of complex networks. Science. 2002; 298(5594):824-7. doi: 10.1126/science.298.5594.824.

Ravasz E, Somera AL, Mongru DA, Oltvai ZN, Barabási AL. Hierarchical organization of modularity in metabolic networks. Science. 2002; 297(5586):1551-5. doi: 10.1126/science.1073374.

Bascompte J. Disentangling the web of life. Science. 2009; 325(5939):416-9. doi: 10.1126/science.1170749.

Alon U. Network motifs: theory and experimental approaches. Nat Rev Genet. 2007; 8(6):450-61. doi: 10.1038/nrg2102.

Kashtan N, Itzkovitz S, Milo R, Alon U. Efficient sampling algorithm for estimating subgraph concentrations and detecting network motifs. Bioinformatics. 2004; 20(11):1746-58. doi: 10.1093/bioinformatics/bth163.

Tarazona S, Furió-Tarí P, Turrà D, Pietro AD, Nueda MJ, Ferrer A, et al. Data quality aware analysis of differential expression in RNA-seq with NOISeq R/Bioc package. Nucleic Acids Res. 2015; 43(21):e140.  doi: 10.1093/nar/gkv711.

Jia C, Kong X, Koltes JE, Gou X, Yang S, Yan D, et al. Gene Co-Expression Network Analysis Unraveling Transcriptional Regulation of High-Altitude Adaptation of Tibetan Pig. PLoS One. 2016; 11(12):e0168161. doi: 10.1371/journal.pone.0168161.
